# Metabolic Response of “*Candidatus* Accumulibacter Phosphatis” Clade II C to Changes in Influent P/C Ratio

**DOI:** 10.3389/fmicb.2016.02121

**Published:** 2017-01-05

**Authors:** Laurens Welles, Ben Abbas, Dimitry Y. Sorokin, Carlos M. Lopez-Vazquez, Christine M. Hooijmans, Mark C. M. van Loosdrecht, Damir Brdjanovic

**Affiliations:** ^1^Department of Environmental Engineering and Water Technology, UNESCO-IHE Institute for Water EducationDelft, Netherlands; ^2^Department of Biotechnology, Delft University of TechnologyDelft, Netherlands; ^3^Winogradsky Institute of Microbiology, Research Center of Biotechnology, Russian Academy of Sciences (RAS)Moscow, Russia

**Keywords:** polyphosphate-accumulating organisms (PAO), glycogen-accumulating organisms (GAO), phosphate limitation, metabolic response, microbial population dynamics

## Abstract

The objective of this study was to investigate the ability of a culture highly enriched with the polyphosphate-accumulating organism, “*Candidatus* Accumulibacter phosphatis” clade IIC, to adjust their metabolism to different phosphate availabilities. For this purpose the biomass was cultivated in a sequencing batch reactor with acetate and exposed to different phosphate/carbon influent ratios during six experimental phases. Activity tests were conducted to determine the anaerobic kinetic and stoichiometric parameters as well as the composition of the microbial community. Increasing influent phosphate concentrations led to increased poly-phosphate content and decreased glycogen content of the biomass. In response to higher biomass poly-phosphate content, the biomass showed higher specific phosphate release rates. Together with the phosphate release rates, acetate uptake rates also increased up to an optimal poly-phosphate/glycogen ratio of 0.3 P-mol/C-mol. At higher poly-phosphate/glycogen ratios (obtained at influent P/C ratios above 0.051 P-mol/C-mol), the acetate uptake rates started to decrease. The stoichiometry of the anaerobic conversions clearly demonstrated a metabolic shift from a glycogen dominated to a poly-phosphate dominated metabolism as the biomass poly-phosphate content increased. FISH and DGGE analyses confirmed that no significant changes occurred in the microbial community, suggesting that the changes in the biomass activity were due to different metabolic behavior, allowing the organisms to proliferate under conditions with fluctuating phosphate levels.

## Introduction

To prevent the receiving waters from eutrophication, the Enhanced Biological Phosphorus Removal (EBPR) process is a cost-effective and environmentally-friendly process for phosphorus removal in wastewater treatment activated sludge systems. The organisms responsible for EBPR are called phosphate-accumulating organisms (PAO). Under anaerobic conditions, PAO are able to take up volatile fatty acids (VFA), such as acetate (HAc) and propionate (HPr), and store them intracellularly as poly-β-hydroxyalkanoates (PHA) (Wentzel et al., 1985; Comeau et al., 1986; Mino et al., 1987). The VFA uptake and storage processes require energy and reducing power. For instance, acetate is taken up by active transport, activated to acetyl-CoA, followed by condensation of two acetyl-CoA molecules into acetoacetyl-CoA and subsequently reduced to poly-β-hydroxybutyrate. The required reducing equivalents (NADH) are obtained from glycolysis (Smolders et al., 1994), while required ATP is obtained partly from the glycolysis supplemented by polyphosphate conversion. Cleavage of intracellular polyphosphate (poly-P), and subsequent release of ortho-phosphate into the liquid phase, is assumed the main pathway for all energy generation. Poly-P is converted by either the combined action of polyphosphate: AMP phosphotransferase [(poly-P)n +AMP ➔ (poly-P)n-1 + ADP] and adenylate kinase [2 ADP ➔ ATP + AMP)] or the action of polyphosphate kinase [(poly-P)n + ADP ➔ (poly-P)n-1 + ATP] (Van Groenestijn et al., 1987; Van Niel et al., 1998; Martín et al., 2006; Wilmes et al., 2008). Release of orthophosphate is considered to potentially generate a proton motive force through a PIT system (Van Veen et al., 1994; Saunders et al., 2007; Burow et al., 2008). Glycolysis of intracellular glycogen is considered the main reducing power source (Mino et al., 1987; Smolders et al., 1994), besides this process generates additional energy. However, several studies have shown that glycolysis in PAO can also function as the main energy generating pathway when the energy production pathway from poly-P is limiting (Brdjanovic et al., 1998; Hesselman et al., 2000; Erdal et al., 2008; Zhou et al., 2008; Acevedo et al., 2012; Welles et al., 2014). Under aerobic conditions, PAO are able to grow and to take up and store ortho-phosphate in excess as intracellular polyphosphate, leading to P-removal from the bulk liquid by wastage of activated sludge (Mino et al., 1998).

The stoichiometry and kinetic rates of EBPR anaerobic conversions (HAc + Poly-P + Gly ➔ PHB/PHV + CO2 + ortho-P) are still controversial. Reported anaerobic P-release/HAc-uptake ratios range from 0.15 up to 0.93 P-mol/C-mol (Wentzel et al., 1987; Smolders et al., 1994; Pereira et al., 1996; Hesselman et al., 2000; Kisoglu et al., 2000) and kinetic rates range from 1 up to 7 [C-mmol HAc/(gVSS.h)] (Smolders et al., 1994; Liu et al., 1997; Sudiana et al., 1999; Filipe et al., 2001; Schuler and Jenkins, 2003b). Such differences have often been explained based on differences on microbial composition and operational conditions. Presence of glycogen-accumulating organisms (GAO) has been suggested as a microbial factor affecting the anaerobic stoichiometry (Mino et al., 1987). GAO compete with PAO for substrate but neither release phosphate anaerobically nor store it under aerobic conditions. Besides GAO, the enrichment of different PAO clades is another factor since PAO I mainly relies on poly-P as energy source for VFA uptake, while PAO II utilizes a mixed PAO-GAO metabolism where glycogen generates a significant part of the energy required for VFA uptake (Welles et al., 2015b). Regarding the operational conditions, pH, nature of the carbon source, sodium and calcium concentration affect the anaerobic stoichiometry (Satoh et al., 1992; Smolders et al., 1994; Oehmen et al., 2005; Barat and van Loosdrecht, 2006; Barat et al., 2008; Welles et al., 2014).

In addition, it has been suggested that the PAO biomass P-content affects the anaerobic stoichiometry. In this regard, at short-term, enriched PAO cultures can shift from a poly-P dependent toward a glycogen dependent metabolism when the biomass P-content decreases (Brdjanovic et al., 1998; Hesselman et al., 2000; Erdal et al., 2008; Zhou et al., 2008; Acevedo et al., 2012). Using highly enriched PAO I and II cultures, Welles et al. (2015b) demonstrated that the poly-P depletion led to a seven and two-fold decrease in the anaerobic kinetic rates of PAO I and II, respectively. Although these studies clearly indicated that the PAO possess metabolic flexibility, the results only represent the short-term response to changes in the biomass P-content. Furthermore, in most of the studies the specific PAO clades were either not reported or the biomass consisted of a mixture of different PAO clades (Brdjanovic et al., 1998; Hesselman et al., 2000; Zhou et al., 2008; Erdal et al., 2008; Acevedo et al., 2012). Finally, not all studies covered a broad range of different biomass P-contents (Welles et al., 2015b). Therefore, it remains unclear how the kinetic rates and stoichiometry of specific PAO clades I and II are affected by a wide range of different biomass P-contents during long-term operation.

In two long-term studies, Liu et al. (1997) and Schuler and Jenkins (2003a) observed a gradual shift from a PAO metabolism to a GAO metabolism when the P-influent concentrations and intracellular poly-P content decreased. In particular, Schuler and Jenkins (2003b) observed higher HAc-uptake rates when the metabolism shifted from a GAO- to a PAO-metabolism. To explain their observations, Liu et al. (1997) suggested that a PAO-GAO competition might have taken place; whereas, Schuler and Jenkins (2003a,b) suggested that the PAO and GAO metabolisms could be either two unique metabolisms in separate groups of organisms or two components of one metabolism in one single group of organisms. In those studies; however, no microbial identification analyses were performed. Kong et al. (2002), in a similar study, observed a shift from *Betaproteobacteria* (the subdivision to which most “*Candidatus* Accumulibacter phosphatis” belong) at a high P/C influent ratio to *Alphaproteobacteria* (the subdivision to which “*Defluviicoccus*” belong) and *Gammaproteobacteria* (the subdivisions to which most “*Candidatus* Competibacter phosphatis” belong) at a low P/C influent ratio. Recent studies have demonstrated that PAO I and II have very different characteristics in terms of morphology, stoichiometry and kinetic rates, the ability to denitrify and possibly tolerance to stress conditions as well (Carvalho et al., 2007; Flowers et al., 2009; Slater et al., 2010; Welles et al., 2015b). These differences may lead to the prevalence of specific clades under certain conditions and consequently differences in the metabolic conversions. Considering the metabolic differences, prevalence of specific clades may significantly affect EBPR processes. For instance, differences in the anaerobic stoichiometry may affect the P-release efficiency in combined chemical and biological P-removal and P-recovery processes. Therefore it becomes important to study the metabolism of the specific clades separately to get a better understanding about the conditions favoring the specific clades. Furthermore, considering the large differences in kinetic rates (four times higher for PAO II at poly-P depleted conditions), the different PAO clades can no longer be considered as one organism in modeling approaches (Welles et al., 2015b).

Therefore, the objective of this study was to assess at long-term how the anaerobic kinetics and stoichiometry of a highly enriched PAO II culture are affected by a wide range of different biomass P-contents. This will provide a more quantitative insight into the relationship between storage polymers and anaerobic metabolic pathways, contributing to explain the wide range of different P/HAc ratios and kinetic rates observed in previous studies. From a practical perspective, this important understanding will ultimately help to improve the existing metabolic models, leading to better design and operation of the EBPR processes and, in particular, of combined chemical and biological phosphorus removal and recovery systems where the P-contents in general are lower.

## Materials and methods

### PAO enrichment and SBR operation

The PAO culture was enriched as described in previous studies (Welles et al., 2014, 2015a,b) in a 2.5 L double-jacketed laboratory sequencing batch reactor (SBR). The SBR was operated and controlled automatically in a sequential mode by an Applikon ADI controller also used for data acquisition and storage (e.g., pH and O_2_) using BioXpert software (Applikon, The Netherlands, Schiedam). The reactor was inoculated with activated sludge from a municipal wastewater treatment plant with a 5-stage Bardenpho configuration (Hoek van Holland, The Netherlands).

The SBR was operated in cycles of 6 h (2.25 h anaerobic, 2.25 aerobic and 1.5 settling phase) following similar operating conditions used in previous studies (Smolders et al., 1994; Brdjanovic et al., 1997; Welles et al., 2014). The pH was maintained at 7.0 by dosing 0.4 M HCl and 0.4 M NaOH and temperature was controlled at 20 ± 1°C. Each cycle started with a 5 min sparging phase with nitrogen gas at a flow rate of 30 L/h to create anaerobic conditions. After the first 5 min, 1.25 L of synthetic substrate was fed to the SBR over a period of 5 min and nitrogen gas sparging continued throughout the anaerobic phase. In the aerobic phase, compressed air was sparged to the SBR at a flow rate of 60 L/h. Mixing was provided at 500 rpm, except during settling and decant phases when mixing was switched off.

The SBR was controlled at a biomass retention time (SRT) of 8 days, not taking into account the potential loss of solids in the effluent during removal and biofilm removal during regular cleaning. At the end of the settling period, the supernatant was pumped out from the reactor, leaving 1.25 L of mixed liquor in the reactor. This resulted in a total hydraulic retention time (HRT) of 12 h.

### Experimental phases and medium

To investigate the effect of the storage polymers on the on the kinetic rates and stoichiometry of the anaerobic conversions, six long-term experimental phases were designed with the aim to obtain an EBPR biomass with different P-contents. The SBR was fed with the same medium in all the experimental phases. The only difference was the orthophosphate concentration. The respective orthophosphate concentrations (provided with the addition of NaH_2_PO_4_.H_2_O) are shown in Table 1.

**Table 1:** **Phosphate concentrations and influent P/C ratios applied in the experimental phases**.

**Experimental phases**	**Phosphate concentration**	**Influent P/C ratio**
	**(P-mmol/L)**	**(P-mol/C-mol)**
Phase 0	0.64	0.051
Phase 1	0.48	0.038
Phase 2	0.96	0.076
Phase 3	1.44	0.114
Phase 4	0.48	0.038
Phase 5	0.64	0.051

The concentrated medium was prepared with demineralised water. In the beginning of every cycle, 250 mL of concentrated substrate together with 1000 mL demi water were fed to the reactor. After dilution, the influent contained per liter: 860 mg CH3COONa·3H_2_O (12.6 C-mmol/L, 405 mg COD/L), 107 mg NH_4_Cl (2 N-mmol/L), 120 mg MgSO_4_.7H_2_O, 14 mg CaCl_2_.2H_2_O, 48 mg KCl, 2 mg of allyl-N-thiourea (ATU) to inhibit nitrification, 0.3 mL/L trace element solution, and a defined concentration of NaH_2_PO_4_.H_2_O different in each experimental phase as previously described. The trace element solution was prepared as described by Smolders et al. (1994). Prior to use, both concentrated solutions were autoclaved at 110°C for 1 h. In phase 0, the biomass culture was enriched, which required a long operation time to obtain a high grade enrichment culture dominated by a specific PAO clade. This phase was not continuously monitored. In phase 1, multiple experiments were done to characterize the biomass performance and microbial community in short-term experiments (Welles et al., 2015b). The remaining phases were about 2-3 SRT each with the aim to be long enough for obtaining stable performance and short enough to avoid gradual changes in the microbial community structure.

### SBR monitoring

The performance of the SBR was regularly monitored by measuring ortho-phosphate (${\text{PO}}_{4}^{3-}$-P), acetate (HAc), total suspended solids (TSS) and volatile suspended solids (VSS). Stable performance in the reactor was confirmed by daily observation of the aforementioned parameters as well as by the pH and DO online data. At the end of each experimental phase, (except for phase 0), a cycle test was conducted to determine the anaerobic stoichiometric and kinetic parameters. In the cycle tests, polyhydroxyalkanoate (PHA) and glycogen concentrations were determined in addition to the above described parameters. Furthermore, the composition of the microbial community was characterized by fluorescence *in situ* hybridization (FISH) analysis and denaturing gradient gel electrophoresis (DGGE) analysis.

### Kinetic rates and stoichiometric values

The PO_4_-release rates and HAc-uptake rates were determined using the PO_4_ and HAc profiles observed in the cycle tests and expressed as maximum active biomass specific rates as described by Smolders et al. (1994) and Brdjanovic et al. (1997). For the maintenance activity (in the absence of HAc), only phosphate release was determined and no glycogen consumption. In order to see potential changes in the glycogen content due to maintenance activity, that are significant enough to be measured, different type of tests would need to be conducted (Zeng et al., 2003). As the maintenance activity was not the main focus of this study, no additional tests were conducted. The stoichiometric parameters of interest were: P/HAc, PHV/HAc, PHB/HAc, PHV/PHB, gly/HAc and gly/PHB.

### Analyses

Determination of TSS, VSS and ${\text{PO}}_{4}^{3-}$-P concentrations were performed in accordance with Standard Methods (A.P.H.A., 1995). HAc was determined using a Varian 430-GC Gas Chromatograph (GC) equipped with a split injector (split ratio 1:10), a WCOT Fused Silica column with a FFAP-CB coating (25 m × 0.53 mm × 1 μm), and coupled to a FID detector. Helium gas was used as carrier gas. Temperature of the injector, column and detector were 200°, 105°, and 300°C, respectively. PHB and PHV contents of freeze dried biomass were determined by gas chromatography after a digestion, esterification and extraction step following the method described by Smolders et al. (1994). Glycogen content of freeze dried biomass was determined by HPLC after digestion according to the method described by Smolders et al. (1994) and Dircks et al. (2001) but with an extended digestion of 5 h in 5 mL 0.9 M HCl, using 5 mg of freeze-dried biomass as described by Lanham et al. (2012).

### Characterization of microbial populations

An estimation of the biomass fractions of the populations of interest (PAO Type I, PAO type II and GAO) was based on FISH analyses, following the procedure described by Winkler et al. (2011). All bacteria were targeted by the EUB338 mix (general bacteria probe) (Amann et al., 1990; Amann, 1995; Daims et al., 1999). “*Candidatus* Accumulibacter phosphatis” and “*Candidatus* Competibacter phosphatis” were targeted by PAOMIX probe (mixture of probes PAO462, PAO651, and PAO846) (Crocetti et al., 2000) and GAOMIX probe (mixture of probes GAOQ431 and GAOQ989) (Crocetti et al., 2002), respectively. PAO I (clade IA and other type I clades) and PAO II (clade IIA, IIC, and IID) were targeted by the probes Acc-1-444 and Acc-2-444 (Flowers et al., 2009), respectively. Hybridized samples were examined with Zeiss Axioplan-2 epifluorescence microscope. The quantification of the PAO and GAO biomass fractions (of the entire bacterial community) and the PAO I and PAO II fractions (of the PAO community) in the biomass was carried out via FISH image analysis in a previous study (Welles et al., 2015b).

To confirm the FISH observations and to identify potential changes in the microbial populations at the sub-clade level, 16S-rDNA-PCR DGGE was applied. Samples were collected at the end of each experimental phase. DNA extraction, PCR amplification, DGGE, band isolation, sequencing and identification of microorganisms were carried out according to the procedures described by Bassin et al. (2011). To double confirm the specific PAO clade, ppk1 gene fragments were recovered and analyzed. A direct PCR was done on the gDNA of the biomass sample from experimental stage 1 using the primers for *Accumulibacter* sp. like bacteria and amplifying the near full length polyphosphate kinase I gene, ACCppk1-254F and ACCppk1-1376R (McMahon et al., 2007). The product from PCR was sequenced using both primers. Both reads were assembled using Codoncode aligner software v4.2.7 (Codoncode corp. USA) and submitted for BLASTn (NCBI) analysis.

### Microscopy

For thin sectioning electron microscopy, the cells were first fixed in 3% (v/v) glutaraldehyde for 1 h on ice, then post-fixed in 1% (w/v) OsO_4_ + 0.5 M NaCl for 3 h at room temperature, washed and stained overnight with 1% (w/v) uranyl acetate, dehydrated in ethanol series and embedded in Epoxy resin. The thin sections were finally stained with 1% lead acetate.

### Active biomass

The active biomass concentration was determined as MLVSS excluding PHB, PHV and glycogen (active biomass = MLVSS – PHB – PHV – glycogen). Unbiodegradable particulate endogenous residue, shown to be another non-active biomass component of the MLVSS (Wentzel et al., 1988, 1989a,b) was neglected for the sake of simplicity, and marginal contribution to the MLVSS in these enrichment cultures. The active biomass concentration was expressed in C-mol units by taking into account the experimentally determined composition of PAO (CH_2.09_O_0.54_N_0.20_P_0.015_) (Smolders et al., 1994).

### Estimation of Poly-P

The concentration of PAO Poly-P was estimated on the basis of the inorganic suspended solids to total suspended solids (ISS/TSS) ratio and confirmed using steady-state mass balances as described in Welles et al. (2015b). Equation 1 was developed using the ISS/TSS ratio of the biomass, assuming that (i) the ISS/TSS ratio associated with active biomass in non-EBPR biomass (ISS_b_) was 0.025 mg ISS/mg TSS (as observed in this study after poly-P depletion), (ii) a poly-P composition of (PO_3_)_3_MgK with a P-content (f_P, ppASH_) of 0.31 mg P/mg ISS and, (iii) negligible chemical precipitation. Equation 2, derived from the steady-state mass balance of phosphorus, assuming that (i) the solids in the effluent were negligible, (ii) a ratio of non poly-P phosphorus per VSS (f_P, bVSS_) equal to the P-content of non-EBPR biomass at around 0.023 mg P/mg VSS (Metcalf and Eddy, 2003) and, (iii) absence of chemical precipitation. A detailed description of the development of the equations can be found in Supplementary file.

(1) $$\begin{array}{rcl} poly-P & = & \left(ISS-\;\frac{f_{ISSb,TSS}}{\left(1-f_{ISSb,TSS}\right)}*VSS\right)*f_{P,ppISS} \end{array}$$

(2) $$\begin{array}{rcl} poly-P & = & \;\frac{SRT}{HRT}*\left(T_{Pi}-T_{Pe}\right)-f_{P,bVSS}*VSS \end{array}$$

where;

VSS: Concentration of volatile suspended solids
ISS: Concentration of inorganic suspended solids
Poly-P: Concentration of poly-phosphate
T_P, i_: concentration of total phosphorus in the influent
T_P, e_: Concentration of total phosphorus in the effluent
f_P, bVSS_**:** Ratio of non poly-P phosphorus per VSS
f_ISSb, TSS:_ ISS/TSS ratio associated with active biomass
f_P, ppISS:_ P-content of poly-P
HRT: Hydraulic retention time
SRT: Solids retention time

## Results

### EBPR performance at different P/C influent ratios

**Figure 1:**
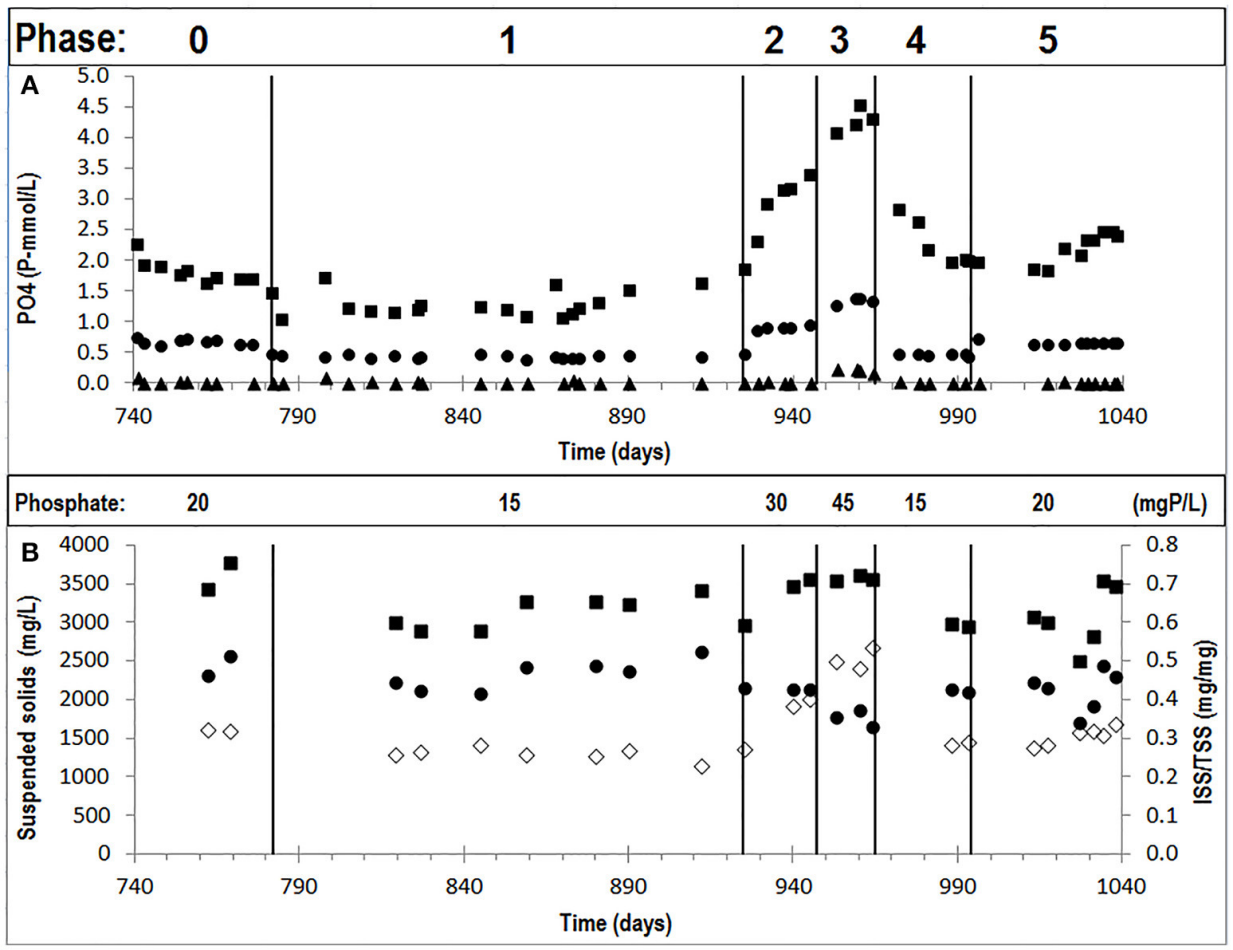
**Concentrations observed in the different cycles of the experimental phases studied: (A)** orthophosphate concentrations in the influent (•), end of the anaerobic phase (■), and end of the aerobic phase (▲); and, **(B)** TSS concentrations in the end of the aerobic phase (■), VSS concentrations in the end of the aerobic phase (•) and ISS/TSS ratios (♢).

An enriched EBPR culture was cultivated with different influent ortho-phosphate concentrations during six experimental phases (phases 0 to 5). The increase in influent phosphate concentration resulted in higher P-release and consequently higher ortho-phosphate concentrations at the end of the anaerobic phase (Figure 1A) and higher biomass ISS/TSS ratio at the end of the aerobic phase (Figure 1B). A decrease in the influent phosphate concentrations decreased both the biomass ISS/TSS ratio content and ortho-phosphate concentrations at the end of the anaerobic phase, showing that the observed patterns were reversible. The P-release/HAc-uptake ratio fluctuated between 0.15 and 0.6 P-mol/C-mol, depending on the influent ortho-phosphate concentration, showing the highest P-release in phase 3 at the highest influent ortho-phosphate concentration. Full P-removal was observed in all phases except in phase 3, leaving an average ortho-phosphate concentration of 0.20 P-mmol/L in the effluent.

### Microbial community at different influent P/C ratios

**Figure 2:**
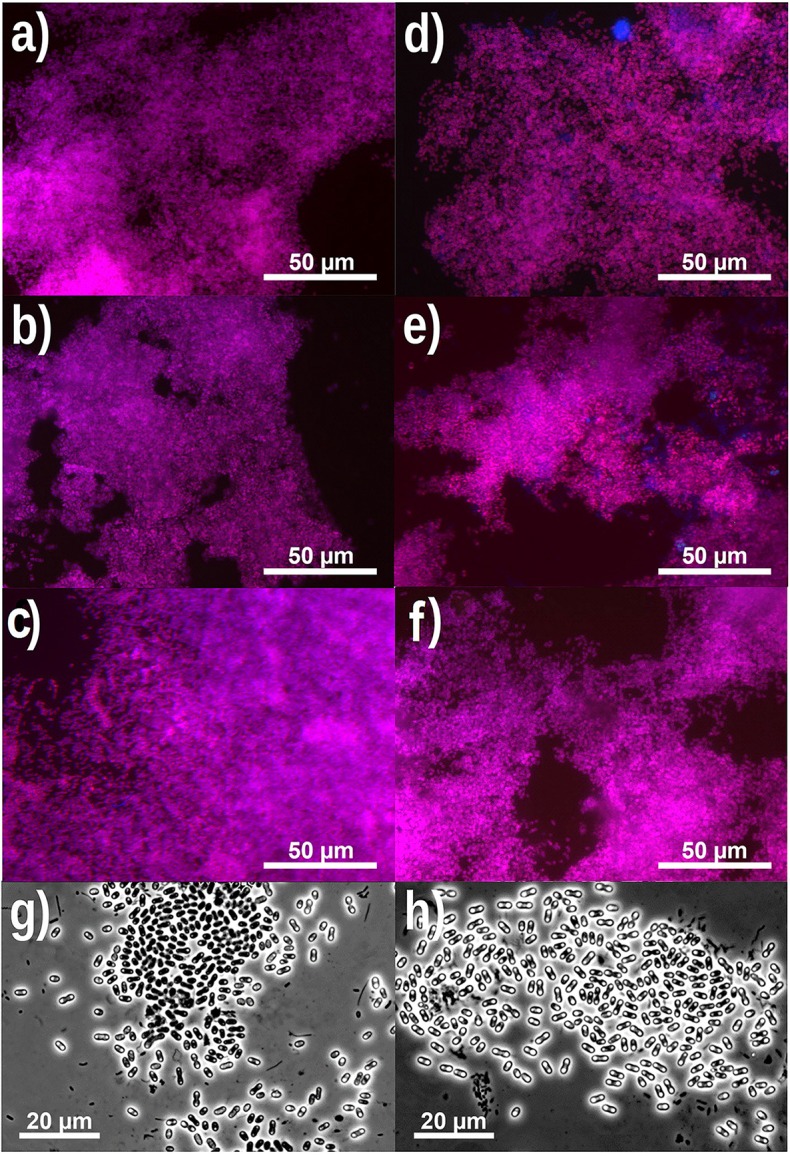
**Representative FISH microscopic images (A–F)** showing the distribution of bacterial populations in biomass samples collected at the end of phase 1 **(A,D)**, phase 3 **(B,E)** and phase 5 **(C,F)** on day 890, 964 and 1034, respectively. In **(A–C)**; blue: EUB mix (Cy5); purple (superposition of blue and red): PAO mix (Cy3); and cyan green (superposition of blue and green): GAO mix (Fluos). In **(D–F)**; blue: PAO mix (Cy5), purple (superposition of blue and red): PAO clade II (Cy3), and cyan green (superposition of blue and green): PAO clade I (Fluos). (For interpretation of the references to color in this figure legend, the reader is referred to the web version of this article.) Phase contrast images **(G,H)** showing the microbial distribution at the end of phase 5.

FISH analysis and quantification showed that PAO were highly dominant in experimental phase 1, 3, and 5 (PAO biomass fraction of 99 ± 3% in phase 1, (Welles et al., 2015b) and more specifically that the PAO population consisted of PAO clade II with minor traces of PAO clade I (biomass fractions of 99 ± 6% PAO II and 1 ± 2% PAO I in phase 1, Welles et al., 2015b) (Figures 2A,D) (raw FISH images are shown in Supplementary Figures 1–24, Supplementary File). GAO were not detected and only minor traces of PAO clade I were observed. In the execution of the FISH microscopic analysis, GAO biomass samples were used as a positive control for the *Competibacter* probes and as negative control for the PAO probes, which confirmed that there were no problems with the probes or analytical procedures to detect GAO presence or PAO absence in the samples. Light microscopy confirmed the dominance of bacteria with the PAO morphology, but also showed that bacteria with smaller dimensions and different morphology were present as well (Figures 2G,H).

**Figure 3:**
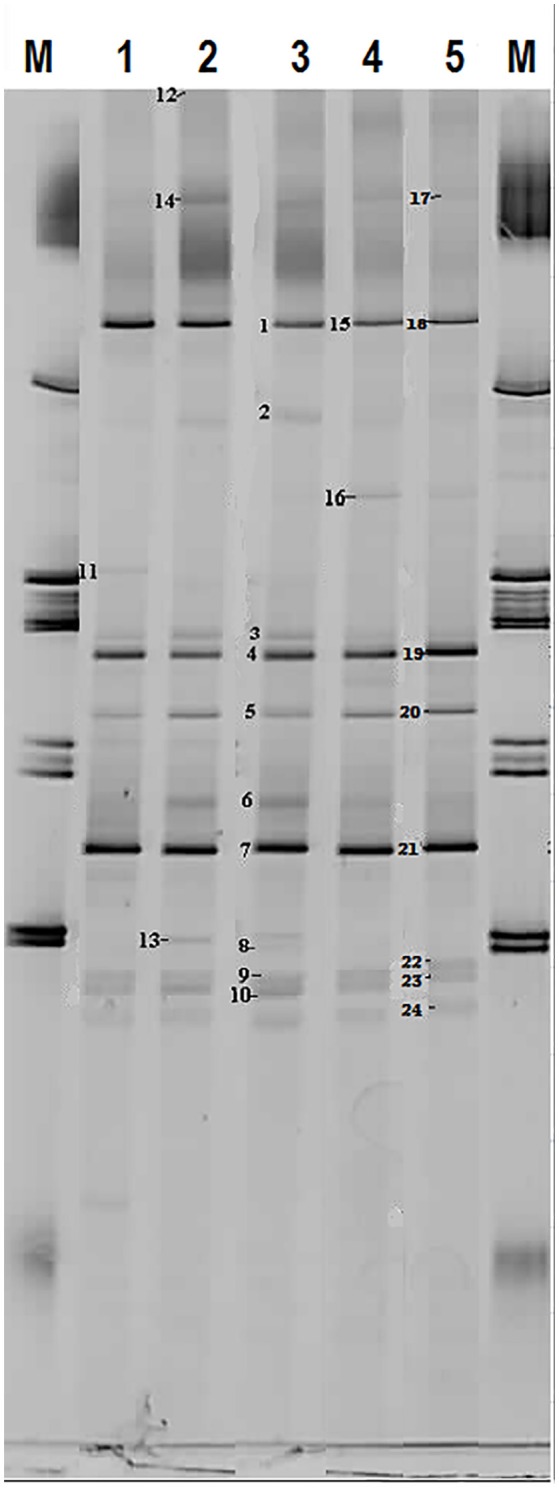
**Microbial identification analysis: 16S rRNA bands obtained by DGGE**. M = marker and Number 1 to 5 correspond to biomass samples collected at the end of the experimental phases on day 890 (phase 1), day 945 (phase 2), day 964 (phase 3), day 993 (phase 4), and day 1034 (phase 5).

**Figure 4:**
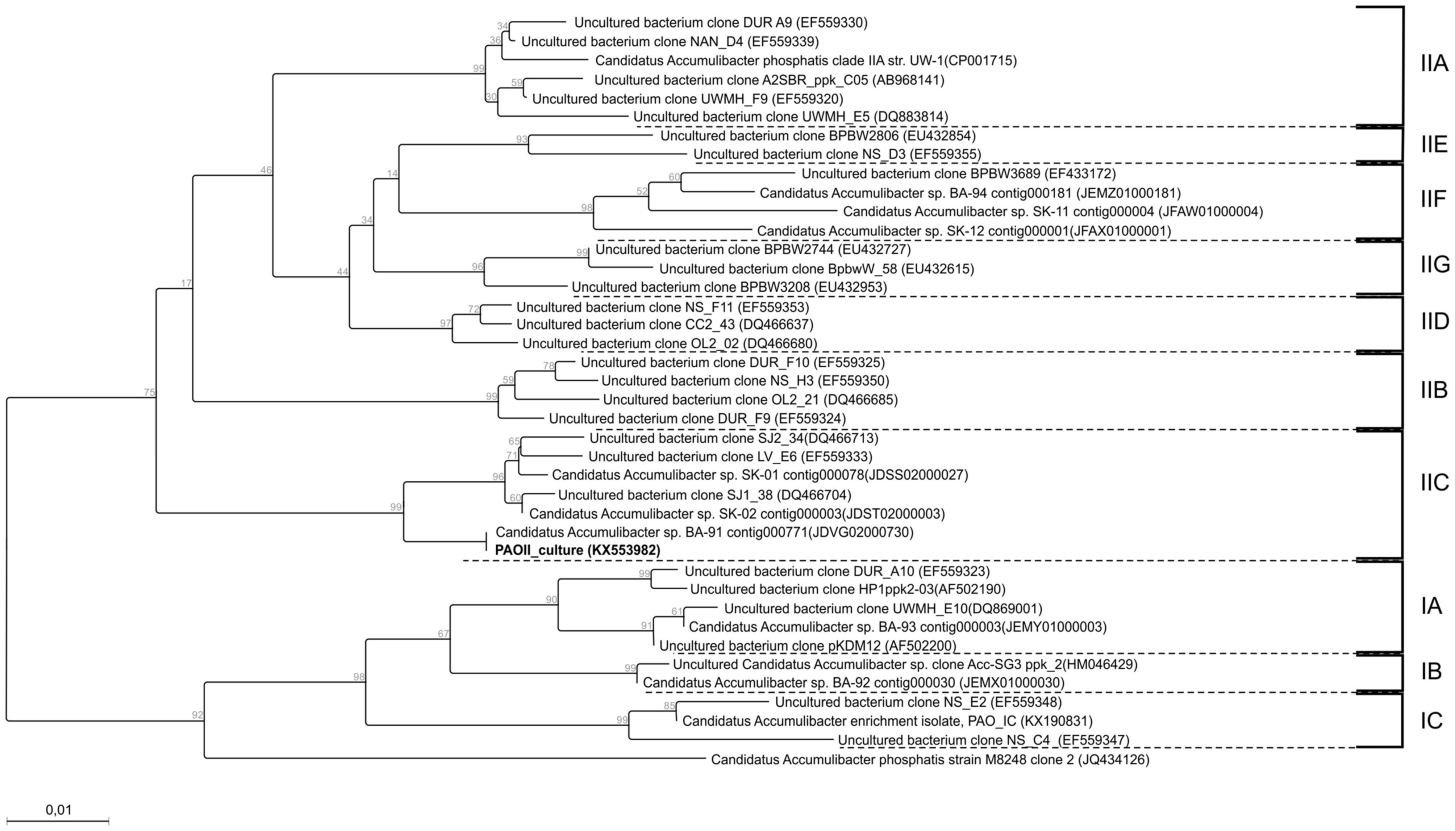
**Phylogenetic tree constructed using the neighbor joining method implemented in the CLC genomic workbench v7.5.1 package**. The distance between sequences were measured using the kimura model. 332 amino-acid positions were used for calculation which correspondsg to a near complete ppk1 gene. The sequence of the ppk1 gene from Rhodocyclus tenuis (AF502199) was used as an outgroup and afterwards pruned from the tree. Also bootstrap (1000 rounds) was performed and the resulting values were displayed on the branching points. All clades are indicated by the text IA till IC and IIA till IIG. The scale bar represent one percent difference in amino-acid composition. The sequence obtained in this study is indicated in bold.

Based on FISH analyses, no significant changes occurred in the relative quantities of PAO I, PAO II, and GAO throughout the execution of the experimental phases (Figures 2B,C,E,F) and therefore quantification was not considered useful in the other experimental phases. 16S-rRNA gene based DGGE profiles were obtained from samples collected at the end of each experimental phase (Figure 3). From the DNA derived DGGE patterns, 24 bands were selected, covering all the unique bands observed in the different phases. The phylogenetic analysis of selected band sequences from all experimental phases was conducted in this study (Table 2). The bacterial groups detected were: (i) *Betaproteobacteria* closely related to “*Candidatus* Accumulibacter phosphatis” (bands 7, 21, and 22); (ii) *Betaproteobacteria* not closely related to “*Candidatus* Accumulibacter phosphatis” (band 9); (iii) *Deltaproteobacteria* (band 13); (iv) *Alphaproteobacteria* (bands 06 and 04); (v) *Armatimonadetes* were also detected (band 20), as well as (vi) bacteria belonging to *Bacteroidetes* (bands 14, 17, 2, 1, 15, 18, 16, 12, 3, and 11). Bands 05, 08, 10, 19, 23, and 24 could not provide sufficient DNA of the required quality for sequencing and the quality of the sequence obtained from band 9 was insufficient for submission to the GenBank A phylogenetic tree analysis of the 16S rRNA-gene sequences obtained from the biomass sample collected at the end of experimental phase 1 was presented in a previous study (Welles et al., 2015b) and revealed that the PAO belonged to “*Candidatus* Accumulibacter phosphatis” clade IIC/D. To confirm the specific PAO clade, the polyphosphate kinase I gene was sequenced and a phylogenetic analysis was performed (Figure 4). This analysis confirmed that the PAO belonged to “*Candidatus* Accumulibacter phosphatis” clade IIC.

**Table 2:** **Microbial identification analysis: phylogenetic analysis of 16S rRNA bands obtained by DGGE**.

**Band**	**Accession no**.	**RDP classification**				**Blast results**		
		**Phylum**	**Class**	**Order**	**Family/(Suborder)**	**Closest relative (excl. uncultured/env. samples)**	**ACC**	**% similarity**
1	KJ395409	Bacteroidetes	Sphingobacteriia	Sphingobacteriales	Chitinophagaceae	Trachelomonas volvocinopsis var. spiralis strain UTEX1313	FJ719709	98
12	KJ395416	Bacteroidetes	Sphingobacteriia	Sphingobacteriales	Chitinophagaceae	Trachelomonas volvocinopsis var. spiralis strain UTEX1313	FJ719709	96
15	KJ395419	Bacteroidetes	Sphingobacteriia	Sphingobacteriales	Chitinophagaceae	Trachelomonas volvocinopsis var. spiralis strain UTEX1313	FJ719709	98
18	KJ395422	Bacteroidetes	Sphingobacteriia	Sphingobacteriales	Chitinophagaceae	Trachelomonas volvocinopsis var. spiralis strain UTEX1313	FJ719709	98
3	KJ395411	Bacteroidetes	Sphingobacteriia	Sphingobacteriales	Chitinophagaceae	Flavihumibacter solisilvae strain 3	KC569790	97
11	KJ395415	Bacteroidetes	Sphingobacteriia	Sphingobacteriales	Chitinophagaceae	Heliimonas saccharivorans strain L2-4	JX458466	87
16	KJ395420	Bacteroidetes	Sphingobacteriia	Sphingobacteriales	Chitinophagaceae	Terrimonas lutea DY	NR_041250	97
2	KJ395410	Bacteroidetes	Flavobacteriia	Flavobacteriales	Flavobacteriaceae	Chryseobacterium sp. Y1D	EU839047	98
14	KJ395418	Bacteroidetes	Flavobacteriia	Flavobacteriales	Flavobacteriaceae	Flavobacterium croceum strain EMB47	NR_043768	99
13	KJ395417	Proteobacteria	Deltaproteobacteria	Myxococcales	(Cystobacterineae)	Cystobacter sp. GNDU S198	KP178619	95
21	KJ395424	Proteobacteria	Betaproteobacteria	Rhodocyclales	Rhodocyclaceae	Candidatus Accumulibacter phosphatis clade IIA str. UW-1	NR_074763	98
22	KJ395425	Proteobacteria	Betaproteobacteria	Rhodocyclales	Rhodocyclaceae	Candidatus Accumulibacter phosphatis clade IIA str. UW-1	NR_074763	95
7	KJ395414	Proteobacteria	Betaproteobacteria	Rhodocyclales	Rhodocyclaceae	Candidatus Accumulibacter phosphatis clade IIA str. UW-1	NR_074763	98
4	KJ395412	Proteobacteria	Alphaproteobacteria	–	–	Rhizobium giardinii CCNWSX1555	KP875539	92
6	KJ395413	Proteobacteria	Alphaproteobacteria	Rhodobacterales	Rhodobacteraceae	Rhodobacter sp. EMB 174	DQ413163	98
20	KJ395423	Armatimonadetes	–	–	–	Fimbriimonas ginsengisolii Gsoil348	CP007139	86

Among the sequenced bands, no “*Candidatus* Competibacter phosphatis” nor *Defluviicoccus* (another known GAO) sequences were detected. There were also no sequences detected that were related to *Gammaproteobacteria*, the subdivision to which “*Candidatus* Competibacter phosphatis” belong. In the different experimental phases, no differences were observed in the dominant bands (band 1, 4, and 7), representing bacteria closely related to *Bacteroidetes*, “*Candidatus* Accumulibacter phosphatis” and *Alphaproteobacteria*, respectively. From the minor bands, band 3, representing *Bacteroidetes* (closely related to *Flavihumibacter petaseus*) and band 6, representing an *Alphaproteobacteria* (closely related to *Rhodobacter capsulatus*), showed slightly higher intensity during phases 2 and 3 which were the phases with higher influent ortho-phosphate concentrations (0.97 and 1.45 P-mmol/L, respectively), but these bands were not detected when the influent ortho-phosphate concentration decreased in phase 4 and 5 to 0.48 and 0.65 P-mmol/L, respectively.

### Effect of influent P/C ratio on intracellular storage polymers

**Figure 5:**
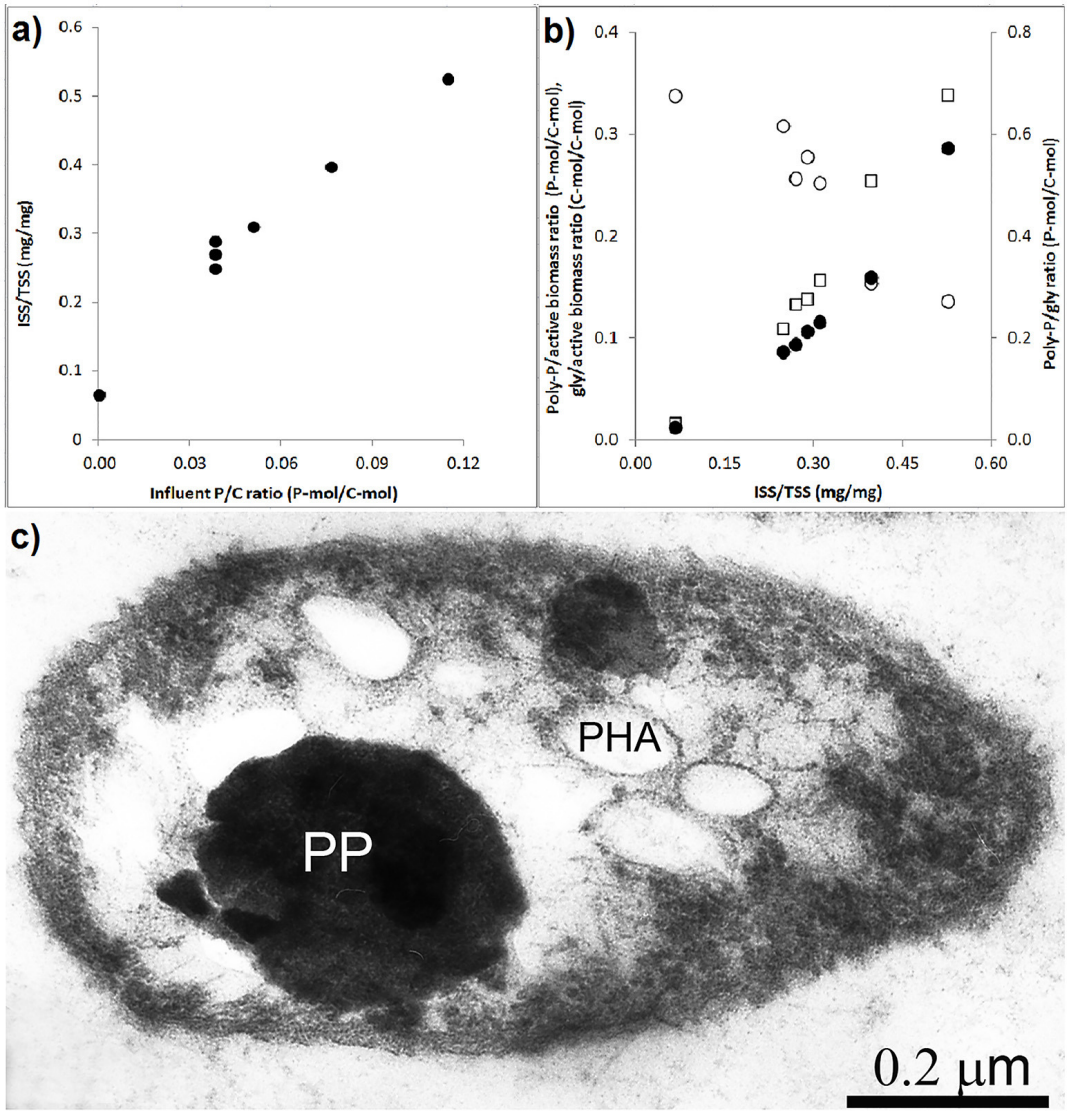
**Storage polymers in enriched PAO biomass samples collected at the end of the experimental phases on day 881 and 890 (phase 1), day 945 (phase 2), day 964 (phase 3), day 993 (phase 4), and day 1034 (phase 5): (A**) ash/TSS ratio as a functions of the influent P/C ratio at the end of the aerobic phase; **(B)** the relationship between the ash/TSS ratio at the end of the aerobic phase and (•) estimated poly-P/active biomass ratio, (○) glycogen/active biomass ratio and (□) poly-P/gly ratio; and, **(C)** electron microscope image of a thin section showing the poly-P, PHA and glycogen organization in the enriched PAO II cell at the end of the anaerobic phase.

The effect of the influent P/C ratio on the ISS/TSS ratio of the biomass and storage polymers is shown in Figures 5A,B. As the influent P/C ratio increased, the ISS/TSS ratio increased linear proportionally. The influent P/C ratio data point that corresponds to zero was obtained in a batch experiment where the poly-P was depleted from the enriched biomass (Welles et al., 2015b). At higher biomass ISS/TSS ratios, the poly-P/active biomass ratio and poly-P/gly ratio increased while the glycogen/active biomass ratio decreased. To get a better understanding of the intracellular organization of storage polymers, thin sections were prepared from the PAO cells taken at the end of the anaerobic phase in experimental phase 2 and analyzed by electron microscopy (Figure 5C). In this phase, with an influent phosphorus concentration of 0.97 P-mmol/L the biomass ISS/TSS ratio reached about 0.4 at the end of the aerobic phase. The microscopy showed that poly-P appeared as 1 or 2 large dense inclusions. Smaller electron transparent inclusions surrounded by a membrane were observed and assumed to correspond to PHA, as it is known to be surrounded by a membrane (Liebergesell et al., 1994; Pieper-Fürst et al., 1994; Steinbüchel et al., 1995; Mayer et al., 1996) while the white dispersed spots were considered to be glycogen, which is known to be freely dispersed in the cytosol (Braña et al., 1980; Kamio et al., 1981).

### Effect of P-content on PAO kinetics

**Figure 6:**
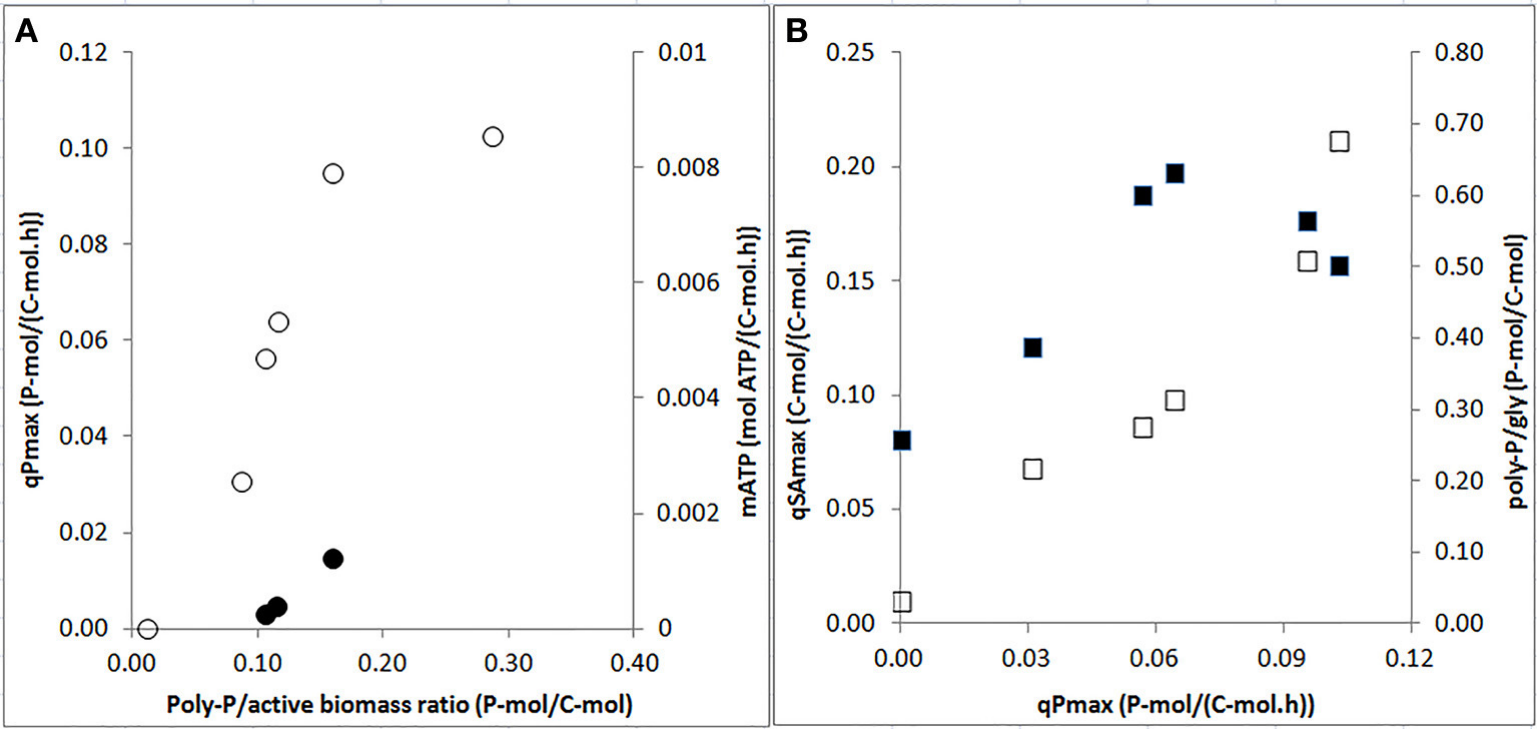
**Effects of poly-P contents and P-release rates on specific biomass kinetic rates at the end of the experimental phases on day 881 and 890 (phase 1), day 945 (phase 2), day 964 (phase 3), day 993 (phase 4), and day 1034 (phase 5): (A)** specific P-release rates during HAc uptake (○: qPmax) and endogenous maintenance activity (•: mATP) as a function of the poly-P/active biomass ratio; and, **(B)** HAc-uptake rates (■: qSAmax) and poly-P/gly ratio (□: poly-P/gly) vs. P-release rates.

The specific P-release rates for acetate uptake increased strongly when the poly-P/active biomass ratio increased from 0 to 0.2 P-mol/C-mol, above which the rates seemed to level off (Figure 6A). Also, the endogenous P-release rate increased with the increasing poly-P/active biomass ratio. When the P-release rate increased, the HAc-uptake rate also increased. A maximum HAc-uptake rate of 0.20 C-mol/C-mol.h was observed when the P-release rate reached 0.07 P-mol/C-mol.h at a poly-P/gly ratio of around 0.3 P-mol/C-mol (Figure 6B). Above this poly-P/gly ratio, the HAc-uptake rate decreased.

### Effect of P-content on PAO stoichiometry

**Figure 7:**
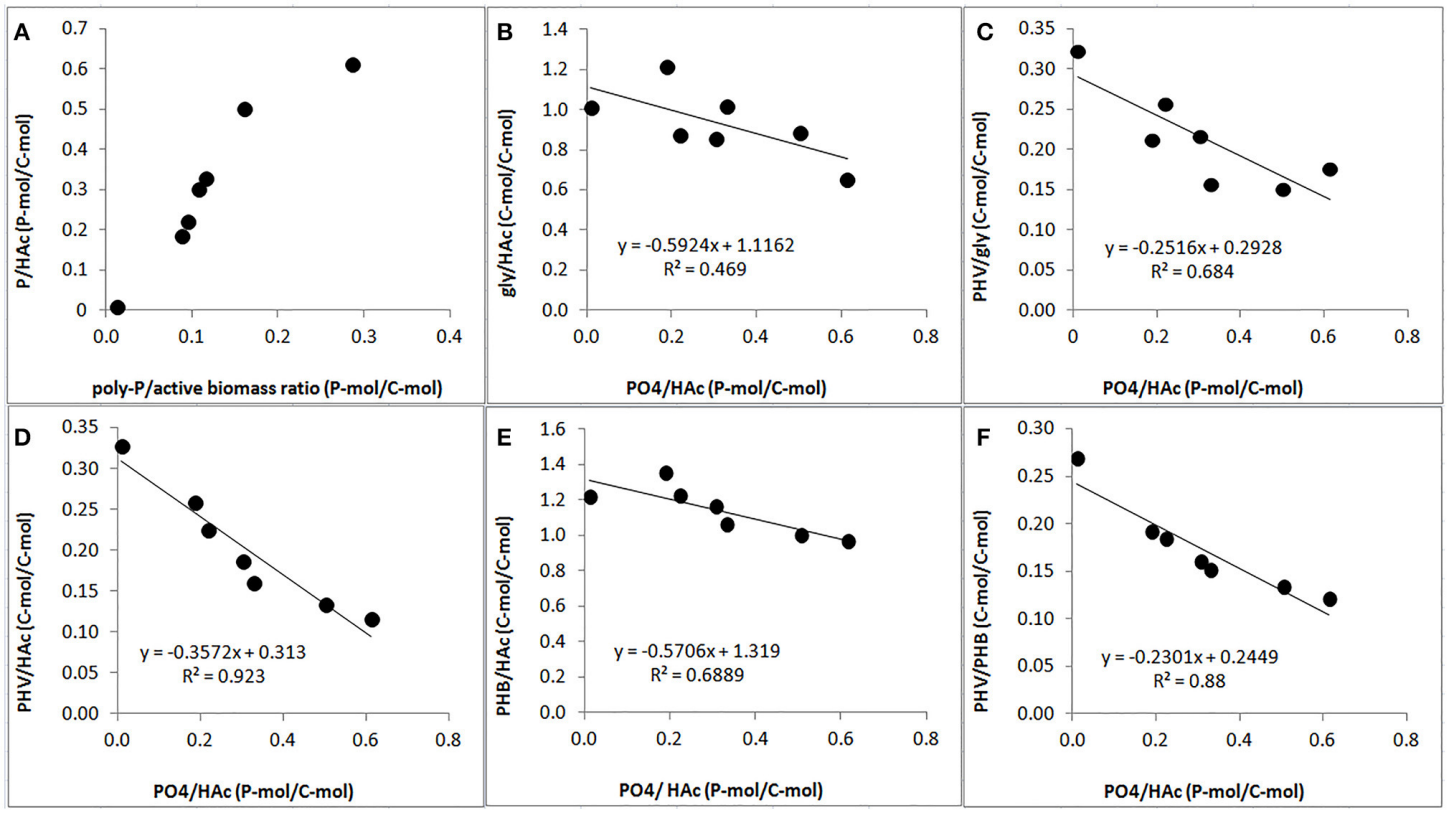
**Effect of poly-P content on anaerobic PAO stoichiometric parameters at the end of the experimental phases on day 881 and 890 (phase 1), day 945 (phase 2), day 964 (phase 3), day 993 (phase 4), and day 1034 (phase 5): (A)** correlation of PO4/HAc ratio (filled circle) and estimated poly-P/active biomass ratio; **(B)** correlation of gly/HAc ratio (filled circle) and PO4/HAc ratio; **(C)** correlation of PHV/gly (filled circle) and PO4 /HAc ratio; **(D)** correlation of PHV/HAc (filled circle) and PO4/HAc ratio; **(E)** correlation of PHB/HAc ratio (filled circle) and PO4/HAc ratio; and, **(F)** correlation of PHV/PHB ratio (filled circle) and PO4/HAc ratio.

A clear relationship was observed between the P-content and stoichiometry of the anaerobic conversions. The PO_4_-release/HAc-uptake ratio increased drastically when the poly-P/active biomass ratio increased (Figure 7A). Consequently, the gly/HAc ratio, PHV/gly ratio, PHV/HAc ratio, PHB/HAc ratio and PHV/PHB ratio decreased when the PO_4_/HAc ratio increased (Figures 7B–F).

## Discussion

### Effect of influent P/C ratio on the microbial population, storage polymers and EBPR performance

FISH analysis showed in experimental phase 1, that the population was highly dominated by “*Candidatus* Accumulibacter phosphatis” clade II. In a previous study (Welles et al., 2015b), with the same biomass culture (SBR-L) in the same experimental phase, the dominance of *Betaproteobacteria* (subdivision to which most “*Candidatus* Accumulibacter phosphatis” belong) was double confirmed by an additional FISH analysis, confirming that the well-known GAO, “*Candidatus* Competibacter phosphatis” and *Defluviicoccus* (belonging to *Gammaproteobacteria* and *Alphaproteobacteria*, respectively) were not present in detectable quantities. In addition, a poly-P staining confirmed that practically all bacteria contained poly-P and thus had the PAO morphology (Welles et al., 2015b). Phylogenetic analysis of the 16S rRNA gene in a previous study (Welles et al., 2015b) and the ppk1 gene analysis in this study, confirmed that the PAO enriched was clade IIC. As FISH analysis demonstrated that the biomass was highly dominated by PAO II and both FISH and DGGE analysis demonstrated that in the different experimental phases, the composition of the microbial community did not change over time, further quantification by FISH microscopy in each experimental phase was not considered useful.

As the poly-P content of the biomass increased, the glycogen content decreased, indicating that poly-P is the preferred storage polymer by PAO IIC which results in higher poly-P/glycogen ratios when the influent phosphate concentrations becomes less limiting at higher influent P/HAc ratios. Still glycogen formation was never completely eliminated. When the poly-P content increased, the metabolism shifted from a mixed PAO-GAO metabolism to a typical PAO metabolism, i.e., P-release/HAc-uptake ratio higher than 0.5 P-mol/C-mol in experimental phase 3. In this experimental phase, the biomass culture was able to remove about 1.45 P-mmol/L, resulting in high poly-P contents (0.54 mg ISS/mg TSS, 0.32 mg P/mg VSS). This removal capacity and poly-P contents are in the range of the highest contents reported in literature (Wentzel et al., 1988; Schuler and Jenkins, 2003a), confirming that the culture was highly enriched with PAO.

While FISH analysis demonstrated that a major fraction of the biomass comprised of PAO (99%), The DGGE profiles showed in addition to the “*Candidatus* Accumulibacter phosphatis” clade IIC/D band two other intense bands belonging to *Bacteroidetes* and *Alphaproteobacteria*. This discrepancy may be explained by the differences in the analytical methods. While the intensity of the DGGE bands represents the quantity of PCR amplified 16s rRNA gene products from specific micro-organisms, the bacterial fractions determined by FISH quantification represent the relative surface areas of bacterial flocs in which the target 16S rRNA is present. A more detailed microscopic analysis of the PAO II dominated flocs (Figures 2G,H) showed that other bacteria of a smaller size were homogeneously enmeshed in each PAO cluster. The smaller bacteria were on a volume or surface area basis present as a minor population (hardly observed by FISH), but their presence was more abundant on a cell-counting basis. In the DGGE analysis the DNA is extracted from the biomass. Considering the large difference in size of the bacteria (about one order of magnitude) and the fact that each bacteria has a genome, the fraction of extracted template DNA belonging to *Bacteroidetes* and *Alphaproteobacteria* in comparison to the fraction of extracted template DNA belonging to PAO may have been much larger than the dry weight biomass fractions. Other factors that may have contributed to the differences in the bacterial quantities obtained from FISH and DGGE data between these organisms may have been: (i) differences in DNA extraction efficiency, (ii) different copy numbers of the 16S rRNA gene in the genomes (only 2 for “*Candidatus* Accumulibacter phosphatis”) and, (iii) differences in PCR amplification efficiency of the 16S rRNA genes. In the perception of the authors, FISH microscopy is more reliable to quantify the bacterial fractions in the microbial community, while DGGE is more reliable for identification of the specific microorganisms. Therefore, the microbial populations other than “*Candidatus* Accumulibacter phosphatis” clade IIC, observed by DGGE, were considered as minor populations, which were not responsible for the major function of the biomass.

### Effect of storage polymers on kinetic rates and stoichiometry

**Figure 8:**
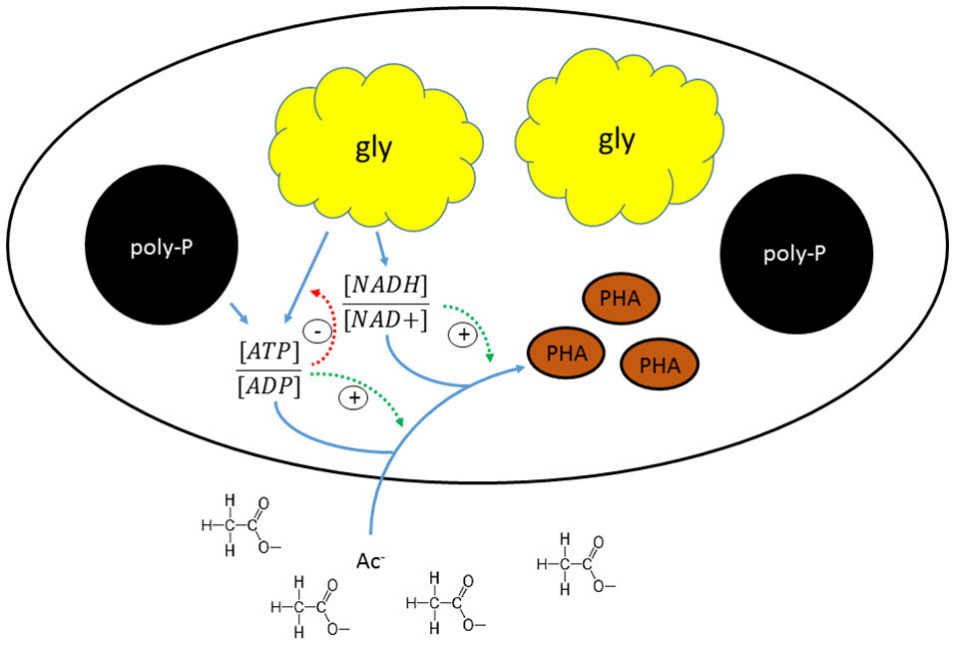
**Schematic representation of the mechanism regulating the metabolic shift in “***Candidatus*** Accumulibacter phosphatis” clade IIC**.

The shift in the stoichiometry of the anaerobic conversions for HAc-uptake indicated that the changes in kinetic P-release and HAc-uptake rates were associated with a shift in the relative ratio of the metabolic fluxes from the different energy generating pathways (glycogen conversion and poly-P degradation), which may have been triggered by a change in the level of the different intracellular storage polymers. A schematic overview of the proposed mechanism that regulates the metabolic shift is shown in Figure 8. When HAc uptake took place to a major extent through a glycogen dependent metabolism in experimental phase 1, the estimated available intracellularly stored poly-P (5.1 P-mmol/L) was about four times higher than the poly-P actually used for HAc uptake (1.3 P-mmol/L). This indicates that the gradual shift in the metabolism of PAO is not driven by a stoichiometric limitation of the available poly-P. Considering that poly-P was stored as large inclusions (Figure 5C), the rate of poly-P consumption may have been poly-P surface area related. This has also been seen in past studies for PHA (Smolders et al., 1995; Murnleitner et al., 1996). Thus, when the poly-P content of the biomass is low, possibly the rate of energy production from poly-P consumption is limited, which then needs to be topped up by energy production from glycogen conversion. The consumption of glycogen allows the cell to harvest 0.5 mol ATP/C-mol glycogen (Zeng et al., 2003), while poly-P conversion generates 1 mol ATP/P-mol poly-P (Van Groenestijn et al., 1987; Smolders et al., 1994).

When the P-release rates increased, the HAc-uptake rates increased up to a maximum value above which the HAc-uptake rate started to decrease again. The increase in the HAc-uptake rate coupled to an increase in the P-release rate suggests that the energy production could have been the rate limiting step for HAc-uptake and that at high poly-P content the energy production from poly-P was faster than the energy production from glycogen. Differences in the energy production rates from poly-P and glycogen may be explained by differences in the number of metabolic conversions of each energy generating pathway. The metabolic pathway for ATP production using poly-P requires only two biochemical conversions and subsequent release of ortho-phosphate over the membrane accompanied by counterions (Van Groenestijn et al., 1987; Saunders et al., 2007). Although the exact pathway for energy production from glycogen remains unclear, such as the type of glycolytic pathways, it probably requires at least 10 biochemical conversions (Satoh et al., 1994) to convert glycogen into PHA and avoid the net-production of reduction equivalents. When the biomass P-content becomes high, the P-release and its associated energy production rate from poly-P conversion become high as well. However, at high biomass P-content, the glycogen content appears to be low, although glycogen consumption for anaerobic substrate uptake was never eliminated in the anaerobic conversions and therefore seems to be essential for the production of reduction equivalents, which was also observed in the study of Schuler and Jenkins (2003a). Possibly the production rate of reduction equivalents is also surface area related. Therefore, a decrease in the glycogen content may trigger a transition where the rate limiting step of the HAc-uptake process changes from the energy production rate to the production rate of reduction equivalents. Thus, at a high biomass P-content, the HAc uptake rate starts to decrease possibly due to limited supply rate of reduction equivalents by glycogen. An optimal HAc uptake rate seems to occur at a poly-P/gly ratio of 0.3 P-mol/C-mol, which corresponds with an ISS/TSS ratio of 0.3 mg ash/mg TSS obtained at an influent P/C ratio of 0.05 P-mol/C-mol. This is roughly the influent P/C ratio at which the PAO culture was originally enriched in this study.

The anaerobic P-release rate for maintenance energy production (determined in the absence of acetate) seemed to also increase when the biomass P-content increased. Assuming that the maintenance coefficient (biomass specific energy requirements per unit of time) of a microorganism is more or less constant under defined environmental conditions (Herbert, 1958; Pirt, 1965), the drastic change in the P-release rate for maintenance energy production suggests that for the production of maintenance energy also a change in metabolism occurred. Possibly, the changes in the P-release rates for anaerobic maintenance energy production may have been compensated by changes in glycogen consumption rates. This hypothesis could have only been verified by additional tests with a longer duration as the potential glycogen consumption for maintenance energy production is very little and the glycogen measurements are not very accurate (Zeng et al., 2003). Due to potential changes in the long term experiments, the prolonged maintenance tests were not performed.

### Assessment of the anaerobic stoichiometric parameters against values reported in literature

Table 3 shows a comparison between the anaerobic kinetic rates and stoichiometric parameters obtained in this study and previously reported values (Liu et al., 1997; Schuler and Jenkins, 2003a,b; among others with highly enriched PAO I, PAO II, and GAO cultures). Similar to the findings of Liu et al. (1997) and Schuler and Jenkins (2003b), the P/HAc stoichiometry increased when the biomass P-content increased but the dependency of the stoichiometry was different. In this study, the P/HAc stoichiometry at a medium initial estimated P/TSS ratio of 0.07 mg/mg was 0.17 P-mol/C-mol against 0.32 P-mol/C-mol reported by Schuler and Jenkins (2003a). This discrepancy can be explained by the findings of a recent study that showed at a de fined poly-P/VSS ratio (0.125 mg/mg), that the anaerobic P/HAc stoichiometry of PAO I was reported to be 2.4 times higher than the stoichiometry of PAO II (Welles et al., 2015b). Possibly the biomass enriched by Schuler and Jenkins (2003a,b,c) may have been dominated by PAO I.

**Table 3:** **Comparison of kinetic and stoichiometric values obtained in this study and previous studies with enriched EBPR cultures**.

**References**	**(Suspected) organisms PAO I, PAO II, GAO**	**SRT**	**HRT**	**P/C influent ratio**	**Initial ISS/TSS**	**Poly-P/active biomass^a^**	**Pns/TSS^b^**	**pH**	**Ca^2+^**	**PHV/PHB**	**PHV/HAc**	**PHB/HAc**	**P/HAc**	**Gly/HAc**	**${\text{q}}_{\text{S}\text{A},\text{ana}}^{\text{M}A\text{X}}$**	**${\text{q}}_{\text{P},\text{ana}}^{\text{M}A\text{X}}$**	**${\text{q}}_{\text{S}\text{A},\text{ana}}^{\text{M}A\text{X}}$**	**${\text{q}}_{\text{P},\text{ana}}^{\text{M}A\text{X}}$**
		**(d)**	**(h)**	**(P-mol/C-mol)**	**(mg/mg)**	**(P-mol/C-mol)**	**(mg/mg)**		**(mg/L)**	**(C-mol/C-mol)**	**(C-mol/C-mol)**	**(C-mol/C-mol)**	**(P-mol/C-mol)**	**(C-mol/C-mol)**	**[C-mmol/(gVSS.h)]**	**[P-mmol/(gVSS.h)]**	**[C-mmol/(C-mol.h)]**	**[P-mmol/(C-mol.h)]**
This study	PAO II	NA	NA	0	0.07	0.01	ND (0.02)	7 ± 0.1	3.8	0.27	0.32	1.22	0.01	1.01	2.3	NA	81	0
	PAO II	8	12	0.038	0.27	0.09	0.069	7 ± 0.05	3.8	0.19	0.26	1.35	0.19	1.22	3.5	0.9	121	31
	PAO II	8	12	0.038	0.29	0.11	0.072	7 ± 0.05	3.8	0.16	0.19	1.17	0.30	0.86	5.5	1.7	188	56
	PAO II	8	12	0.051	0.31	0.12	0.095	7 ± 0.05	3.8	0.15	0.16	1.07	0.33	1.02	6.0	1.9	198	64
	PAO II	8	12	0.077	0.40	0.16	0.130	7 ± 0.05	3.8	0.14	0.14	1.00	0.50	0.89	5.8	3.1	177	95
	PAO II	8	12	0.11	0.53	0.29	0.171	7 ± 0.05	3.8	0.12	0.12	0.97	0.61	0.66	4.9	3.2	157	103
Liu et al., 1997	NA	8	6	0.078	NA	NA	0.13	7 ± 0.1	7.6	NA	NA	NA	0.66	0.42	6.0	3.5	NA	NA
	NA	8	6	0.038	NA	NA	0.08	7 ± 0.1	7.6	NA	NA	NA	0.46	0.65	3.5	1.5	NA	NA
	NA	8	6	0.008	NA	NA	0.02	7-8	7.6	NA	NA	NA	0.02	1.37	1.0	0.01	NA	NA
Schuler and Jenkins, 2003a,b	NA	4	12	0.003	NA	NA	0.018	7.15-7.25	16	NA	NA	NA	0.11	1.19	1.1^d^	NA	NA	NA
	NA	4	12	0.006	NA	NA	0.036	7.15-7.25	16	NA	NA	NA	0.27	0.61	2.5^d^	NA	NA	NA
	NA	4	12	0.012	NA	NA	0.051	7.15-7.25	16	NA	NA	NA	0.31	0.78	2.8^d^	NA	NA	NA
	NA	4	12	0.019	NA	NA	0.082	7.15-7.25	16	NA	NA	NA	0.41	0.62	3.7^d^	NA	NA	NA
	NA	4	12	0.039	NA	NA	0.13	7.15-7.25	16	NA	NA	NA	0.60	n.d	5.4^d^	NA	NA	NA
	NA	4	12	0.062	NA	NA	0.16	7.15-7.25	16	NA	NA	NA	0.73	0.30	6.5^d^	NA	NA	NA
	NA	4	12	0.085	NA	NA	0.14	7.15-7.25	16	NA	NA	NA	0.73	0.33	6.5^d^	NA	NA	NA
	NA	4	12	0.105	NA	NA	0.16	7.15-7.25	16	NA	NA	NA	0.71	0.42	6.5^d^	NA	NA	NA
Welles et al., 2015b	PAO I	8	12	0.65	0.42	NA	0.14	7 ± 0.1	3.8	0.07	0.09	1.27	0.64	0.29	5.3	NA	179	NA
	PAO I	NA	NA	0.03	0.05	NA	ND (0.02)	7 ± 0.1	3.8	0.33	0.37	1.09	0.02	1.28	0.73	NA	23	NA
	PAO II	8	12	0.3	0.25	NA	0.068	7 ± 0.1	3.8	0.19	0.23	1.24	0.22	0.96	4.5	NA	154	NA
	PAO II	NA	NA	0.02	0.07	NA	ND (0.02)	7 ± 0.1	3.8	0.27	0.32	1.19	0.01	0.98	2.3	NA	80	NA
Zeng et al., 2003	GAO	6.6	8	0.005	0.03	NA	NA	7 ± 0.1	6.8	0.38	0.52	1.39	NA	1.20	NA	NA	170	NA
Lopez-Vazquez et al., 2007^c^	GAO	10	12	0.005	0.10	NA	NA	7 ± 0.1	3.8	0.1	0.69	1.28	0.01	1.20	NA	NA	200	NA

*NA, not applicable; ND, not detected (with the used FISH probes); $q_{SA,ana}^{MAX}$, maximum specific HAc-uptake rate; $q_{P,ana}^{MAX}$, maximum specific P-release rate*.

a *Calculated with equation 1 in material and methods*.

b *Calculated with equation 2 in material and methods*.

c *Stoichiometric values obtained from figures*.

d *Calculated with equation in Figure 3 (Schuler and Jenkins, 2003b) and P/HAc data from Table 3 (Schuler and Jenkins (2003a)*.

### Implications on EBPR performance and modeling

The findings drawn in this study suggest that in wastewaters containing high VFA concentrations from hydrolysis and fermentation processes in the sewerage, the anaerobic kinetic rates of PAO are highly dependent on the poly-P content of PAO. The kinetic rates and associated competitiveness of certain PAO clades, increase as the P-content of the biomass increases. In the anaerobic phase of WWTP's treating such wastewaters, PAO perform their best at a medium range P-content (like observed in this study on PAO IIC) or at a high P-content (Schuler and Jenkins, 2003a,b,c). If, in a certain system, GAO become dominant, the availability of P per PAO biomass, and there with the PAO specific P-content, increases. Consequently, the HAc uptake rate may increase. However, as the P-content increases due to high influent P/C ratios, the return of phosphorus from the sludge line to the water line or if GAO prevail, the aerobic P-uptake ability of PAO is reduced because PAO become saturated with poly-P. In addition, the endogenous P-release activity of PAO increases, which may lead to higher secondary P-release processes in an anaerobic zone that follows the aerated stage. Dependent on different factors such as the climate, the type of sewerage system and the dynamics of wastewater in the sewerage, the hydrolysis and fermentation processes in the sewerage and the associated VFA production, may be limited. In such cases, the PAO in the activated sludge systems may rely for a major extend on the VFA production from fermentation processes in the anaerobic stage of the activated sludge system, which are in general much slower than the VFA consumption processes by PAO I, II and GAO when fed with high VFA concentrations. In such cases, the competition between the different microbial communities will not be determined by the maximum rates of PAO I, II and GAO, but instead the competition between the organisms will be majorly be determined by the Ks values of the VFA uptake processes of the respective microbial communities.

The observed relationship between the anaerobic stoichiometry and the sludge P-content implies that a determination of the anaerobic HAc-uptake stoichiometry at pH 7.0 under strict anaerobic conditions at a temperature of 20°C may help to estimate the poly-P content of the PAO present in the activated sludge. Without any microbial characterization, a stoichiometric P/HAc value in the range of 0.65-0.72 P-mol/C-mol in activated sludge at the end of the anaerobic phase, would indicate that the PAO present in the sludge are saturated with poly-P and that GAO are not present. In such cases, the net ortho-phosphate uptake capacity of the activated sludge may be limited due to incapability of PAO to take up additional phosphate during the anoxic or aerobic phase.

## Author contributions

All authors contributed to the design of the experiments. LW performed the experiments and drafted the manuscript. BA conducted the microbial analysis. All authors critically read and contributed to the final version of the manuscript. All authors read and approved the final manuscript.

## Funding

This research project was financed by UNESCO-IHE internal research fund.

### Conflict of interest statement

The authors declare that the research was conducted in the absence of any commercial or financial relationships that could be construed as a potential conflict of interest.

## References

[B1] Acevedo B. Oehmen A. Carvalho G. Seco A. Borras L. Barat R. (2012). Metabolic shift of polyphosphate-accumulating organisms with different levels of poly-phosphate storage. Water Res. 46, 1889–1900. 10.1016/j.watres.2012.01.003 22297158

[B2] Amann R. I. (1995). *In situ* identification of microorganisms by whole cell hybridization with rRNA-targeted nucleic acid probes, in Molecular Microbial Ecology Manual, eds Akkermans A. D. L. van Elsas J. D. de Bruijn F. J. (London: Kluwer Academic Publisher), 1–15.

[B3] Amann R. I. Binder B. J. Olson R. J. Chisholm S. W. Devereux R. Stahl D. A. (1990). Combination of 16S rRNA-targeted oligonucleotide probes with flow cytometry for analyzing mixed microbial populations. Appl. Environ. Microbiol. 56, 1919–1925. 2200342 10.1128/aem.56.6.1919-1925.1990 PMC184531

[B4] A.P.H.A (1995). Standard Methods for the Examination of Water and Waste Water, 17th Edn. Washington, DC: American Public Health Association.

[B5] Barat R. Montoya T. Borrás L. Ferrer J. Seco A. (2008). Interactions between calcium precipitation and the polyphosphate-accumulating bacteria metabolism. Water Res. 42, 3415–3424. 10.1016/j.watres.2008.05.003 18538819

[B6] Barat R. van Loosdrecht M. C. M. (2006). Potential phosphorus recovery in a WWTP with the BCFS process: interactions with the biological process. Water Res. 40, 3507–3516. 10.1016/j.watres.2006.08.006 17011018

[B7] Bassin J. P. Pronk M. Muyzer G. Kleerebezem R. Dezotti M. van Loosdrecht M. C. M. (2011). Effect of elevated salt concentrations on the aerobic granular sludge process: linking microbial activity with microbial community structure. Appl. Environ. Microbiol. 77, 7942–7953. 10.1128/AEM.05016-11 21926194 PMC3208997

[B8] Braña A. F. Manzanal M. B. Hardisson C. (1980). Occurrence of polysaccharide granules in sporulating hyphae of Streptomyces viridochromogenes. J. Bacteriol. 144, 1139–1142. 7440503 10.1128/jb.144.3.1139-1142.1980 PMC294780

[B9] Brdjanovic D. van Loosdrecht M. C. M. Hooijmans C. M. Alaerts G. J. Heijnen J. J. (1997). Temperature effects on physiology of biological phosphorus removal. J. Environ. Eng-ASCE 123, 144–154. 10.1061/(ASCE)0733-9372(1997)123:2(144)

[B10] Brdjanovic D. van Loosdrecht M. C. M. Hooijmans C. M. Mino T. Alaerts G. J. Heijnen J. J. (1998). Effect of polyphosphate limitation on the anaerobic metabolism of phosphorus-accumulating microorganisms. Appl. Microbiol. Biotechnol. 50, 273–276. 10.1007/s002530051289

[B11] Burow L. C. Mabbett A. N. Blackall L. L. (2008). Anaerobic glyoxylate cycle activity during simultaneous utilization of glycogen and acetate in uncultured Accumulibacter enriched in enhanced biological phosphorus removal communities. ISME J. 2, 1040–1051. 10.1038/ismej.2008.45 18784756

[B12] Carvalho G. Lemos P. C. Oehmen A. Reis M. A. (2007). Denitrifying phosphorus removal: linking the process performance with the microbial community structure. Water Res. 41, 4383–4396. 10.1016/j.watres.2007.06.065 17669460

[B13] Comeau Y. Hall K. J. Hancock R. E. W. Oldham W. K. (1986). Biochemical-model for enhanced biological phosphorus removal. Water Res. 20, 1511–1521. 10.1016/0043-1354(86)90115-6

[B14] Crocetti G. R. Banfield J. F. Keller J. Bond P. L. Blackall L. L. (2002). Glycogen accumulating organisms in laboratory-scale and full-scale wastewater treatment processes. Microbiology 148, 3353–3364. 10.1099/00221287-148-11-3353 12427927

[B15] Crocetti G. R. Hugenholtz P. Bond P. L. Schuler A. Keller J. Jenkins D.. (2000). Identification of polyphosphate-accumulating organisms and design of 16S rRNA-directed probes for their detection and quantitation. Appl. Environ. Microbiol. 66, 1175–1182. 10.1128/AEM.66.3.1175-1182.2000 10698788 PMC91959

[B16] Daims H. Brühl A. Amann R. Amann R. Schleifer K. H. Wagner M. (1999). The domain-specific probe EUB338 is insufficient for the detection of all bacteria: development and evaluation of a more comprehensive probe set. Syst. Appl. Microbiol. 22, 345–352. 10.1016/S0723-2020(99)80053-8 10553296

[B17] Dircks K. Henze M. van Loosdrecht M. C. Mosbæk H. Aspegren H. (2001). Storage and degradation of poly-β-hydroxybutyrate in activated sludge under aerobic conditions. Water Res. 35, 2277–2285. 10.1016/S0043-1354(00)00511-X 11358308

[B18] Ekama G. A. Wentzel M. C. (2004). A predictive model for the reactor inorganic suspended solids concentration in activated sludge systems. Water Res. 38, 4093–4106. 10.1016/j.watres.2004.08.005 15491657

[B19] Erdal U. G. Erdal Z. K. Daigger G. T. Randall C. W. (2008). Is it PAO-GAO competition or metabolic shift in EBPR system? Evidence form an experimental study. Water Sci. Technol. 58, 1329–1334. 10.2166/wst.2008.734 18845874

[B20] Filipe C. D. Daigger G. T. Grady C. P. Jr. (2001). A metabolic model for acetate uptake under anaerobic conditions by glycogen accumulating organisms: Stoichiometry, Kinetics and the effect of pH. Biotechnol. Bioeng. 76, 17–31. 10.1002/bit.1022 11400103

[B21] Flowers J. J. He S. Yilmaz S. Noguera D. R. McMahon K. D. (2009). Denitrification capabilities of two biological phosphorus removal sludges dominated by different ‘Candidatus Accumulibacter’ clades. Environ. Microbiol. Rep. 1, 583–588. 10.1111/j.1758-2229.2009.00090.x 20808723 PMC2929836

[B22] Herbert D. E. N. I. S. (1958). Some principles of continuous culture. Recent Prog. Microbiol. 3, 381–396.

[B23] Hesselman R. P. X. von Rummell R. Resnick S. M. Hany R. Zehnder A. J. B. (2000). Anaerobic metabolism of bacteria performing enhanced biological phosphate removal. Water Res. 34, 3487–3494. 10.1016/S0043-1354(00)00092-0

[B24] Kamio Y. Terawaki Y. Nakajima T. Matsuda K. (1981). Structure of glycogen produced by Selenomonas ruminantium. Agric. Biol. Chem. 45, 209–216. 10.1271/bbb1961.45.209

[B25] Kisoglu Z. Erdal U. Randall C. W. (2000). The effect of COD/TP ratio on intracellular storage materials, system performance and kinetic parameters in a BNR system, in Proceedings of the 73rd Annual Water Environment Federation Technical Exposition and Conference (California, CA).

[B26] Kong Y. H. Beer M. Rees G. N. Seviour R. J. (2002). Functional analysis of microbial communities in aerobic-anaerobic sequencing batch reactors fed with different phosphorus/carbon)P/C) ratios. Microbiology 148, 2299–2307. 10.1099/00221287-148-8-2299 12177324

[B27] Lanham A. B. Ricardo A. R. Coma M. Fradinho J. Carvalheira M. Oehmen A.. (2012). Optiomisation of glycogen quantification in mixed microbial cultures. Biores. Technol. 118, 518–525. 10.1016/j.biortech.2012.05.087 22717572

[B28] Liebergesell M. Sonomoto K. Madkour M. Mayer F. Steinbüchel A. (1994). Purification and characterization of the poly (hydroxyalkanoic acid) synthase from Chromatium vinosum and localization of the enzyme at the surface of poly (hydroxyalkanoic acid) granules. Eur. J. Biochem. 226, 71–80. 10.1111/j.1432-1033.1994.tb20027.x 7957260

[B29] Liu W. T. Nakamura K. Matsuo T. Mino T. (1997). Internal energy-based competition between poly-phosphate- and glycogen-accumulating bacteria in biological phosphorus removal reactors-effect of P/C feeding ratio. Water Res. 31, 1430–1438. 10.1016/S0043-1354(96)00352-1

[B30] Lopez-Vazquez C. M. Song Y. I. Hooijmans C. M. Brdjanovic D. Moussa M. S. Gijzen H. J.. (2007). Short-term temperature effect on the anaerobic metabolism of glycogen accumulating organisms. Biotechnol. Bioeng. 97, 483–495. 10.1002/bit.21302 17171717

[B31] Martín H. G. Ivanova N. Kunin V. Warnecke F. Barry K. W. McHardy A. C.. (2006). Metagenomic analysis of two enhanced biological phosphorus removal (EBPR) sludge communities. Nat. Biotechnol. 24, 1263–1269. 10.1038/nbt1247 16998472

[B32] Mayer F. Madkour M. H. Pieper-Furst U. Wieczorek R. Liebergesell M. Steinbuchel A. (1996). Electron microscopic observations on the macromolecular organization of the boundary layer of bacterial PHA inclusion bodies. J. Gen. Appl. Microbiol. 42, 445–455. 10.2323/jgam.42.445

[B33] McMahon K. D. Yilmaz S. He S. Gall D. L. Jenkins D. Keasling J. D. (2007). Polyphosphate kinase genes from full-scale activated sludge plants. Appl. Microbiol. Biotechnol. 77, 167–173. 10.1007/s00253-007-1122-6 17671784

[B34] Metcalf Eddy Inc. (2003). Wastewater Engineering - Treatment and Reuse, 4th Edn. New York, NY: Mc Graw Hill.

[B35] Mino T. Arun V. Tsuzuki Y. Matsuo T. (1987). Effect of phosphorus accumulation on acetate metabolism in the biological phosphorus removal process, in Biological Phosphate Removal from Wastewaters, Advances in Water Pollution Control, R. Ramadori (Oxford: Pergamon Press), 27–38.

[B36] Mino T. van Loosdrecht M. C. M. Heijnen J. J. (1998). Microbiology and biochemistry of the enhanced biological phosphate removal process. Water Res. 32, 3193–3207. 10.1016/S0043-1354(98)00129-8

[B37] Murnleitner E. Kuba T. van Loosdrecht M. C. M. Heijnen J. J. (1996). An integrated metabolic model for aerobic and denitrifying biological phosphorus removal. Biotechnol. Bioeng. 54, 434–450. 10.1002/(SICI)1097-0290(19970605)54:5<434::AID-BIT4>3.0.CO;2-F 18634136

[B38] Oehmen A. Zeng R. J. Yuan Z. Keller J. (2005). Anaerobic metabolism of propionate by polyphosphate-accumulating organisms in enhanced biological phosphorus removal systems. Biotechnol. Bioeng. 91, 43–53. 10.1002/bit.20480 15880463

[B39] Pereira H. Lemos P. C. Reis M. A. M. Crespo J. P. S. G. Carrondo M. J. T. Santos H. (1996). Model for carbon metabolism in biological phosphorus removal processes based on *in vivo* C13-NMR labelling experiments. Water Res. 30:2128 10.1016/0043-1354(96)00035-8

[B40] Pieper-Fürst U. Madkour M. H. Mayer F. Steinbüchel A. (1994). Purification and characterization of a 14-kilodalton protein that is bound to the surface of polyhydroxyalkanoic acid granules in Rhodococcus ruber. J. Bacteriol. 176, 4328–4337. 10.1128/jb.176.14.4328-4337.1994 8021220 PMC205646

[B41] Pirt S. J. (1965). The maintenance energy of bacteria in growing cultures. Proc. R. Soc. Lond. B. Biol. Sci. 163, 224–231. 10.1098/rspb.1965.0069 4378482

[B42] Satoh H. Mino T. Matsuo T. (1992). Uptake of organic substrates and accumulation of polyhydroxyalkanoates linked with glycolysis of intracellular carbohydrates under anaerobic conditions in the biological excess phosphate removal processes. Water Sci. Technol. 26, 933–942.

[B43] Satoh H. Mino T. Matsuo T. (1994). Deterioration of Enhanced Biological Phosphorus removal by the domination of microorganisms without polyphosphate accumulation. Wat. Sci. Technol. 30, 203–211.

[B44] Saunders A. M. Mabbett A. N. McEwan A. G. Blackall L. L. (2007). Proton motive force generation from stored polymers for the uptake of acetate under anaerobic conditions. FEMS Microbiol. Lett. 274, 245–251. 10.1111/j.1574-6968.2007.00839.x 17610509

[B45] Schuler A. J. Jenkins D. (2003a). Enhanced biological phosphorus removal from wastewater by biomass with different phosphorus contents, part 1: experimental results and comparison with metabolic models. Water Environ. Res. 75, 485–498. 10.2175/106143003X141286 14704008

[B46] Schuler A. J. Jenkins D. (2003b). Enhanced biological phosphorus removal from wastewater by biomass with different phosphorus contents, part 2: anaerobic adenosine triphosphate utilization and acetate uptake rates. Water Environ. Res. 75, 499–511. 10.2175/106143003X141295 14704009

[B47] Schuler A. J. Jenkins D. (2003c). Enhanced biological phosphorus removal from wastewater by biomass with different phosphorus contents, part III: anaerobic sources of reducing equivalents. Water Environ. Res. 75, 512–522. 14704010 10.2175/106143003x141303

[B48] Slater F. R. Johnson C. R. Blackall L. L. Beiko R. G. Bond P. L. (2010). Monitoring associations between clade-level variation, overall community structure and ecosystem function in enhanced biological phosphorus removal (EBPR) systems using terminal-restriction fragment length polymorphism (T-RFLP). Water Res. 44, 4908–4923. 10.1016/j.watres.2010.07.028 20701946

[B49] Smolders G. J. F. Van der Meij J. Van Loosdrecht M. C. M. Heijnen J. J. (1994). Model of the anaerobic metabolism of the biological phosphorus removal process: stoichiometry and pH influence. Biotechnol. Bioeng. 43, 461–470. 10.1002/bit.260430605 18615742

[B50] Smolders G. J. F. van Loosdrecht M. C. M. Heijnen J. J. (1995). A metabolic model for the biological phosphorus removal process. Water Sci. Technol. 31, 79–97. 10.1016/0273-1223(95)00182-M

[B51] Steinbüchel A. Aerts K. Babel W. Föllner C. Liebergesell M. Madkour M. H.. (1995). Considerations on the structure and biochemistry of bacterial polyhydroxyalkanoic acid inclusions. Can. J. Microbiol. 41, 94–105. 10.1139/m95-175 7606669

[B52] Sudiana I. Mino T. Satoh H. Nakamura K. Matsuo T. (1999). Metabolism of enhanced biological phosphorus removal and non-enhanced biological phosphorus removal sludge with acetate and glyucose as carbon source. Water Sci. Technol. 39:29 10.1016/S0273-1223(99)00141-9

[B53] Van Groenestijn J. W. Deinema M. H. Zehnder A. J. B. (1987). ATP production from polyphosphate in *Acinetobacter* strain 210A. Arch. Microbiol. 148, 14–19. 10.1007/BF00429640

[B54] Van Niel E. W. J. Appeldoorn K. J. Zehnder A. J. B. Kortstee G. J. J. (1998). Inhibition of anaerobic phosphate release by nitric oxide in activated sludge. Appl. Environ. Microbiol. 64, 2925–2930. 9687452 10.1128/aem.64.8.2925-2930.1998 PMC106794

[B55] Van Veen H. W. Abee T. Kortstee G. J. Pereira H. Konings W. N. Zehnder A. J. (1994). Generation of a proton motive force by the excretion of metal-phosphate in the polyphosphate-accumulating Acinetobacter johnsonii strain 210A. J. Biol. Chem. 269, 29509–29514. 7961934

[B56] Welles L. Lopez-Vazquez C. M. Hooijmans C. M. Van Loosdrecht M. C. M. Brdjanovic D. (2014). Impact of salinity on the anaerobic metabolism of phosphate-accumulating organisms (PAO) and glycogen-accumulating organisms (GAO). Appl. Microbiol. Biotechnol. 98, 7609–7622. 10.1007/s00253-014-5778-4 24831025

[B57] Welles L. Lopez-Vazquez C. M. Hooijmans C. M. van Loosdrecht M. C. M. Brdjanovic D. (2015a). Impact of salinity on the aerobic metabolism of phosphate-accumulating organisms. Appl. Microbiol. Biotechnol. 99, 3659–3672. 10.1007/s00253-014-6287-1 25524698

[B58] Welles L. Tian W. D. Saad S. Abbas B. Lopez-Vazquez C. M. Hooijmans C. M.. (2015b). Accumulibacter clades Type I. and II performing kinetically different glycogen-accumulating organisms metabolisms for anaerobic substrate uptake. Water Res. 83, 354–366. 10.1016/j.watres.2015.06.045 26189167

[B59] Wentzel M. C. Dold P. L. Ekama G. A. Marais G. (1989b). Enhanced polyphosphate organism cultures in activated sludge systems. Part III: kinetic model. Water, S. A. 15, 89–102.

[B60] Wentzel M. C. Dold P. L. Ekama G. A. Marais G. V. R. (1985). Kinetics of biological phosphorus release. Water Sci. Technol. 17, 57–71.

[B61] Wentzel M. C. Dold P. L. Loewenthal R. E. Ekama G. A. Marais G. V. R. (1987). Experiments towards establishing the kinetics of biological excess phosphorus removal, in Advances in Water Pollution Control: Biological Phosphate Removal from Wastewaters, ed Ramadori R. (Oxford: Pergamon Press), 79–91.

[B62] Wentzel M. C. Ekama G. A. Loewenthal R. E. Dold P. L. Marais G. (1989a). Enhanced polyphosphate organism cultures in activated sludge systems. Part II: experimental behaviour. Water S. A. 15, 71–88.

[B63] Wentzel M. C. Loewenthal R. E. Ekama G. A. Marais G. V. R. (1988). Enhanced polyphosphate organism cultures in activated sludge systems- Part 1: enhanced culture development. Water S.A. 14, 81–92.

[B64] Wilmes P. Andersson A. F. Lefsrud M. G. Wexler M. Shah M. Zhang B.. (2008). Community proteogenomics highlights microbial strain-variant protein expression within activated sludge performing enhanced biological phosphorus removal. ISME J. 2, 853–864. 10.1038/ismej.2008.38 18449217

[B65] Winkler M. K. Bassin J. P. Kleerebezem R. De Bruin L. M. Van den Brand T. P. H. Van Loosdrecht M. C. M. (2011). Selective sludge removal in a segregated aerobic granular biomass system as a strategy to control PAO–GAO competition at high temperatures. Water Res. 45, 3291–3299. 10.1016/j.watres.2011.03.024 21513967

[B66] Zeng R. J. van Loosdrecht M. C. M. Yuan Z. Keller J. (2003). Metabolic model for glycogen-accumulating organisms in anaerobic/aerobic activated sludge systems. Biotechnol. Bioeng. 81, 92–105. 10.1002/bit.10455 12432585

[B67] Zhou Y. Pijuan M. Zeng R. J. Lu H. Yuan Z. (2008). Could polyphosphate-accumulating organisms (PAO) be glyccogen-accumulating organisms (GAO)? Water Res. 42, 2361–2368. 10.1016/j.watres.2008.01.003 18222522

