# Supplementary material for: Metabolic Response of “Candidatus Accumulibacter Phosphatis” Clade II C to Changes in Influent P/C Ratio
Source: Front Microbiol. 2017 Jan 5;7:2121. doi: 10.3389/fmicb.2016.02121 (PMC5216660; doi:10.3389/fmicb.2016.02121)
Supplement: Supplementary file 1 [file Presentation1.pdf]

Supplementary Material

Metabolic response of '*Candidatus* Accumulibacter phosphatis' clade II C to changes in influent P/C ratio

Laurens Welles*, Ben Abbas, Dimitry Y. Sorokin, Carlos M. Lopez-Vazquez, Christine M. Hooijmans, Mark C.M. van Loosdrecht, Damir Brdjanovic

*** Correspondence:** Corresponding Author: email@uni.edu

1 Detailed description of equations for poly-P estimation

Poly-P was estimated on the basis of the ISS/TSS ratio and confirmed using steady-state mass balances as described in Welles et al. (2015). Equation 1 was developed using the ISS/TSS ratio of the biomass, assuming that (i) the ISS/TSS ratio associated with active biomass in non-EBPR biomass (ISS_b) was 0.025 mg ISS/mgTSS (as observed in this study after poly-P depletion), (ii) a poly-P composition of $(PO_3)_3MgK$ with a P-content ($f_{P,ppASH}$) of 0.31mgP/mgISS and (iii) negligible chemical precipitation. Equation 2, derived from the steady-state mass balance of phosphorus, assuming that (i) the solids in the effluent were negligible, (ii) a ratio of non poly-P phosphorus per VSS ($f_{P,bVSS}$) equal to the P-content of non-EBPR biomass at around 0.023 mgP/mgVSS (Metcalf and Eddy, 2003) and (iii) absence of chemical precipitation.

$$ISS_{pp} = ISS - ISS_b \qquad \text{(Eq. 1a)}$$

$$ISS_b = f_{ISSb,TSS,} / (1- f_{ISSb,TSS}) * VSS \qquad \text{(Eq. 1b)}$$

$$ISS_{pp} = ISS - f_{ISSb,TSS,} / (1- f_{ISSb,TSS}) * VSS \qquad \text{(Eq. 1c)}$$

$$\text{poly-P} = ISS_{pp} * f_{P,ppISS} \qquad \text{(Eq 1d)}$$

$$\text{poly-P} = (ISS - f_{ISSb,TSS,} / (1- f_{ISSb,TSS}) * VSS) * f_{P,ppISS} \qquad \text{(Eq. 1e)}$$

$$Pns = \text{poly-P} + P_b \qquad \text{(Eq 2a)}$$

$$P_b = f_{P,bVSS} *VSS \qquad \text{(Eq 2b)}$$

$$d (T_{P,e}*V_p)/dt = Q_i * (T_{P,i} - T_{P,e}) - Q_w * TSS * f_{P,TSS} = 0 \qquad \text{(Eq 2c)}$$

$$Q_i /V_p * (T_{P,i} - T_{P,e}) - Q_w / V_p * TSS * f_{P,TSS} = 0 \qquad \text{(Eq 2d)}$$

$$1/HRT * (T_{P,i} - T_{P,e}) - 1/SRT * TSS * f_{P,TSS} = 0 \qquad \text{(Eq 2e)}$$

$$Pns = SRT/HRT * (T_{P,i} - T_{P,e}) \qquad \text{(Eq 2f)}$$

$poly\text{-}P = SRT/HRT * (T_{P,i} - T_{P,e}) - f_{P,bVSS} * VSS$ (Eq 2g)

where;

TSS: Concentration of total suspended solids

VSS: Concentration of volatile suspended solids

ISS: Concentration of inorganic suspended solids

ISS_b: Concentrations of inorganic suspended solids associated with active biomass

ISS_{pp}: Concentration of inorganic suspended solids associated with poly-P

Pns: concentration of non-soluble total phosphorus

P_b: Concentration of phosphate associated with active biomass

poly-P: poly-phosphate concentration

$T_{P,i}$: Total phosphorus concentration in the influent

$T_{P,e}$: Total phosphorus concentration in the effluent

$f_{P,TSS}$: Ratio of total P per TSS

$f_{P,bVSS}$: Ratio of non poly-P phosphorus per VSS

$f_{ISSb,TSS}$: ISS/TSS ratio associated with active biomass

$f_{P,ppISS}$: P-content of poly-P

V_p: Working volume of reactor

Q_i: Influent flow rate

Q_w: Wastage of activated sludge flow rate

HRT: Hydraulic retention time

SRT: Solids retention time

The ash content associated with active biomass (under poly-P depleted conditions) obtained in this study was comparable with the ash content obtained in the GAO or mixed PAO-GAO cultures enriched in previous studies under similar conditions (Welles et al., 2014; Welles et al., 2015b; Welles et al. 2015c). However, some past studies have reported higher values. In a study by Ekama and Wentzel (2004), a model was developed and calibrated, using data from 22 different studies on 30 aerobic and anoxicï aerobic nitrificationï denitrification systems and 18 anaerobicï anoxicï aerobic ND biological excess P removal systems, variously fed artificial and real wastewater, and from 3 to 20 days sludge age. The study indicated that the ISS/TSS ratio associated to OHO biomass was 0.13.

The significant difference in the ISS/TSS ratios observed in this study and those determined in the study of Ekama and Wentzel (2004), maybe caused by differences in the concentrations of the minerals (for example, in some cases 4x higher Ca^{2+} concentrations) and operational pH values (values up to 7.74) (Wentzel et al., 1989b). This suggests, that the equations provided in this study may only be used in for highly enriched PAO and GAO cultures that are cultivated under similar conditions (medium composition, concentration and pH).

The stoichiometry of the anaerobic release and aerobic uptake of potassium (K^+) magnesium (Mg^{2+}) and calcium (Ca^{2+}) in this study was during the anaerobic and aerobic phase for the K^+/PO_4-P and Mg^{2+}/ PO_4-P ratios: release, 0.227 Kmol/P-mol, 0.302 Mg-mol/P-mol; uptake 0.234 K-mol/P-mol, 0.304

Mg-mol/P-mol. Negligible differences in calcium concentrations were observed, indicating

that Ca^{2+} did not play a significant role in biological or chemical phosphate removal Welles et al. (2015).

On the basis of the measured release of Mg^{2+} and K^+ (Welles et al., 2015), the poly-P content of the biomass in this study was $(PO_3)_3Mg_{0.91}K_{0.69}$. However, as the positive charge balance covered only 83 to 84% of the negative charges from the phosphate units in poly-P, it was assumed that in the bulk of poly-P inclusions contained more K and Mg resulting in the composition $(PO_3)_3MgK$.

2 Raw FISH images

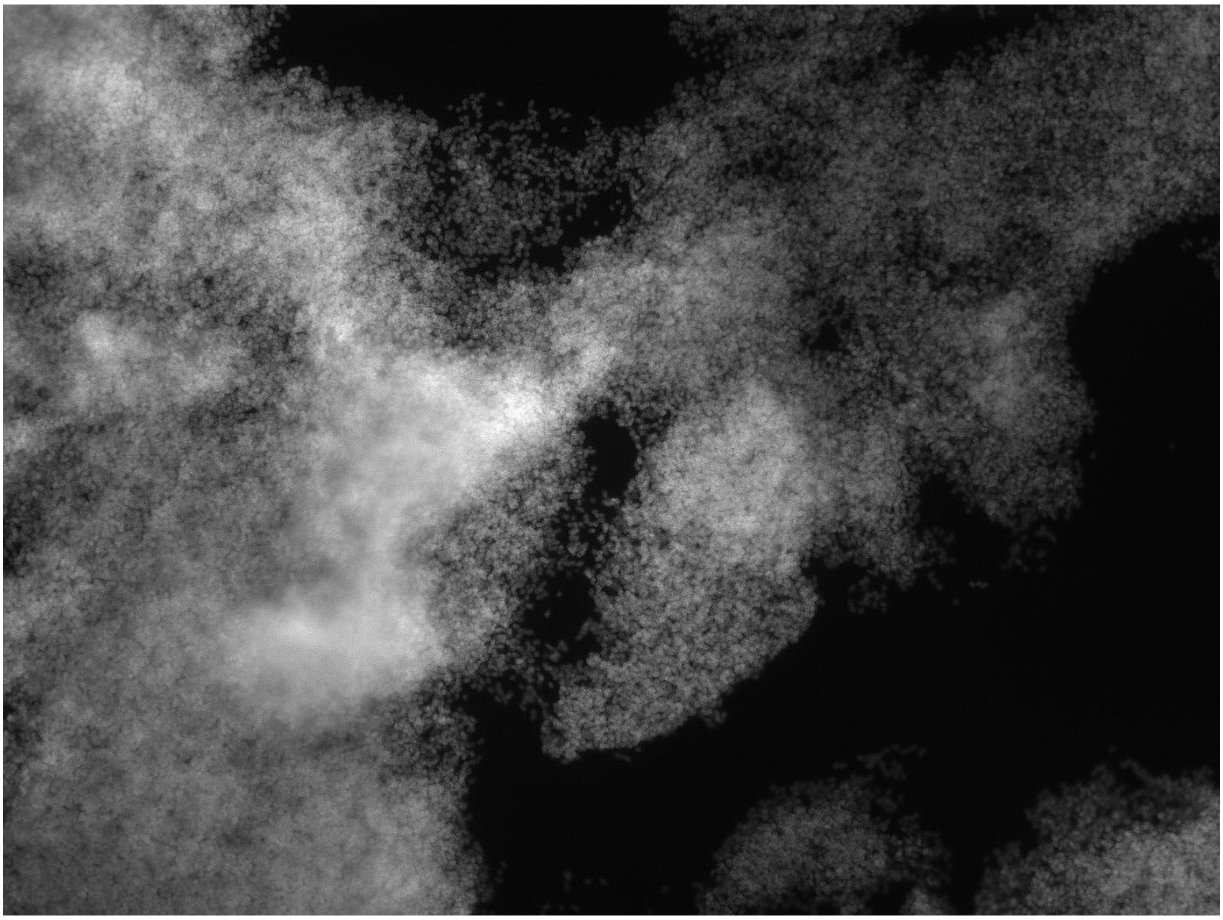

Supplementary Figure 1. Raw FISH image of Figure 2a, day 890, EUB (Cy5).

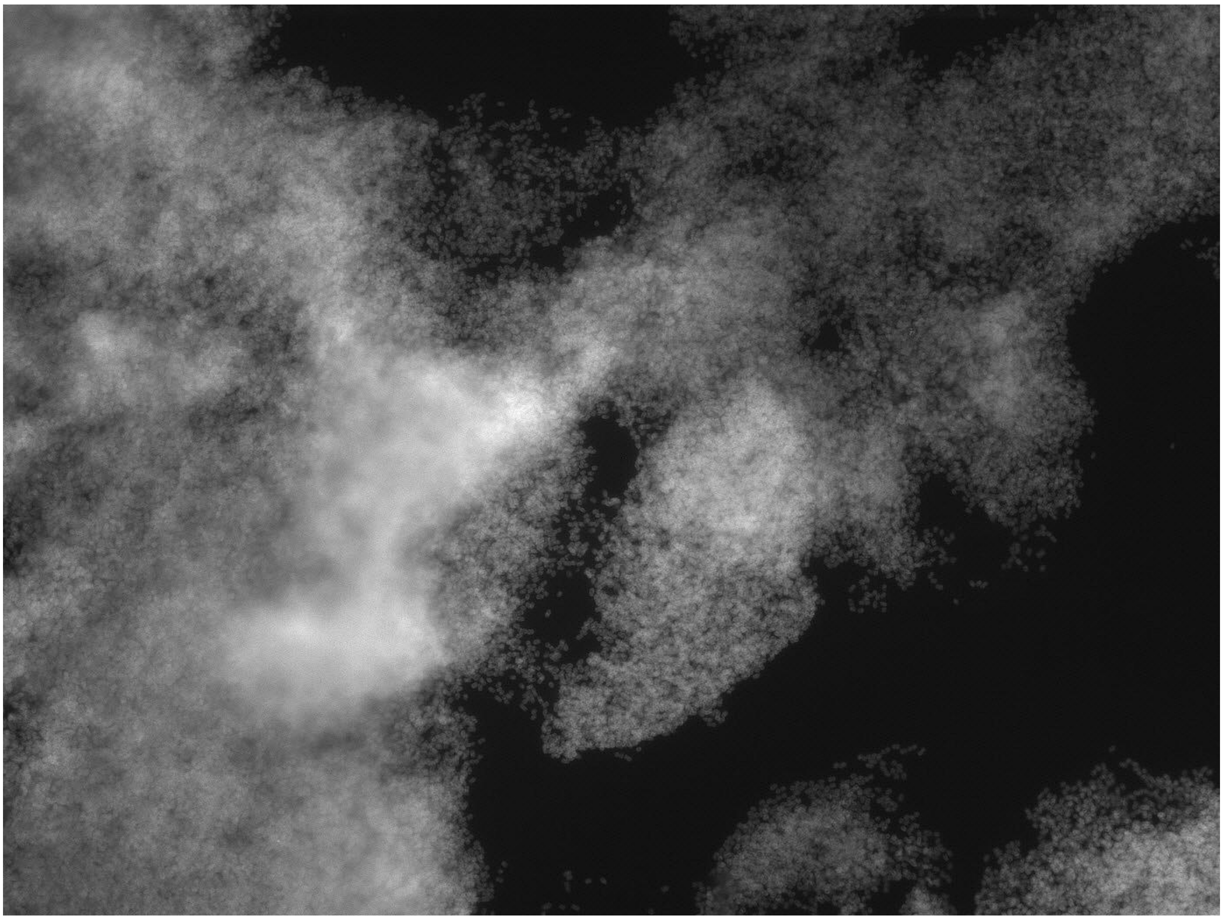

Supplementary Figure 2. Raw FISH image of Figure 2a, day 890, PAOmix (Cy3).

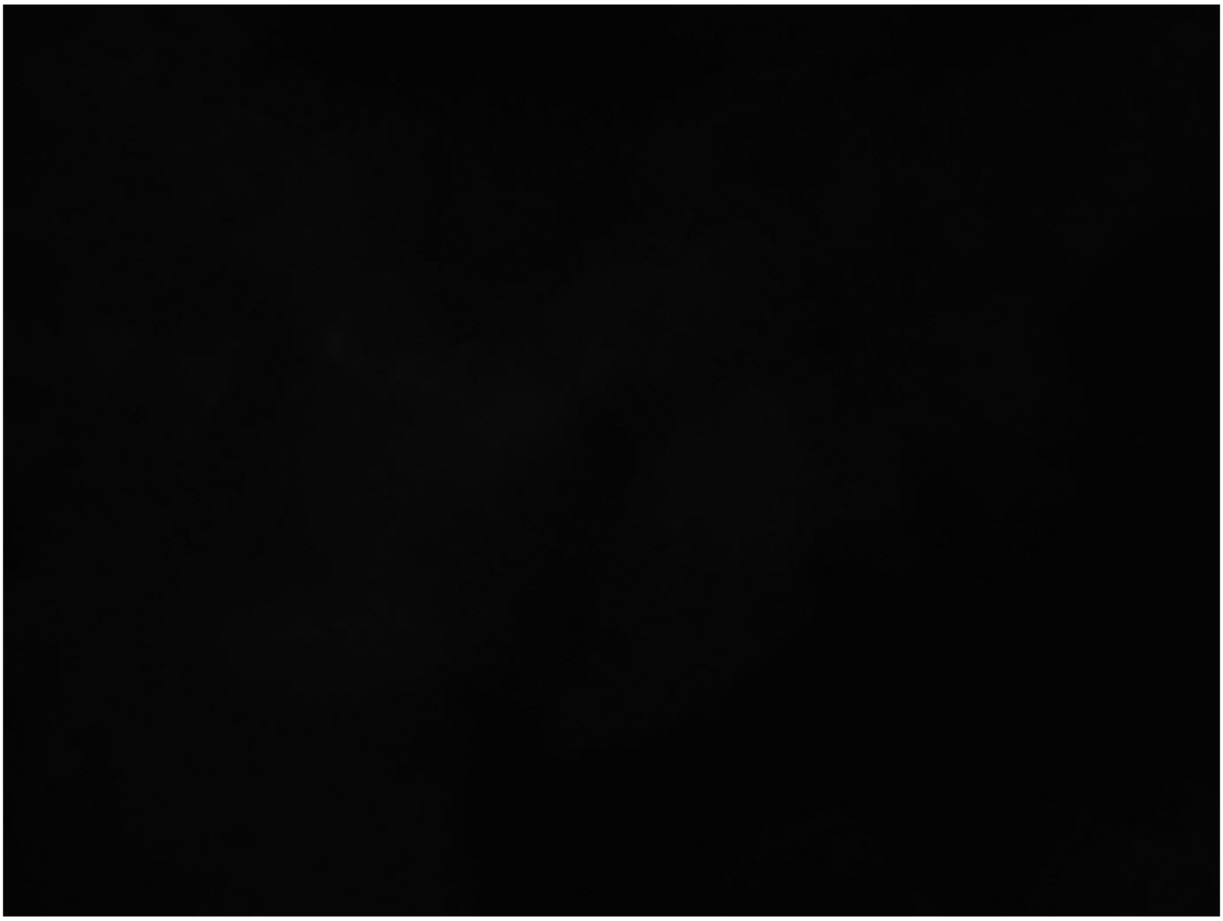

Supplementary Figure 3. Raw FISH image of Figure 2a, day 890, GAOmix (Fluos).

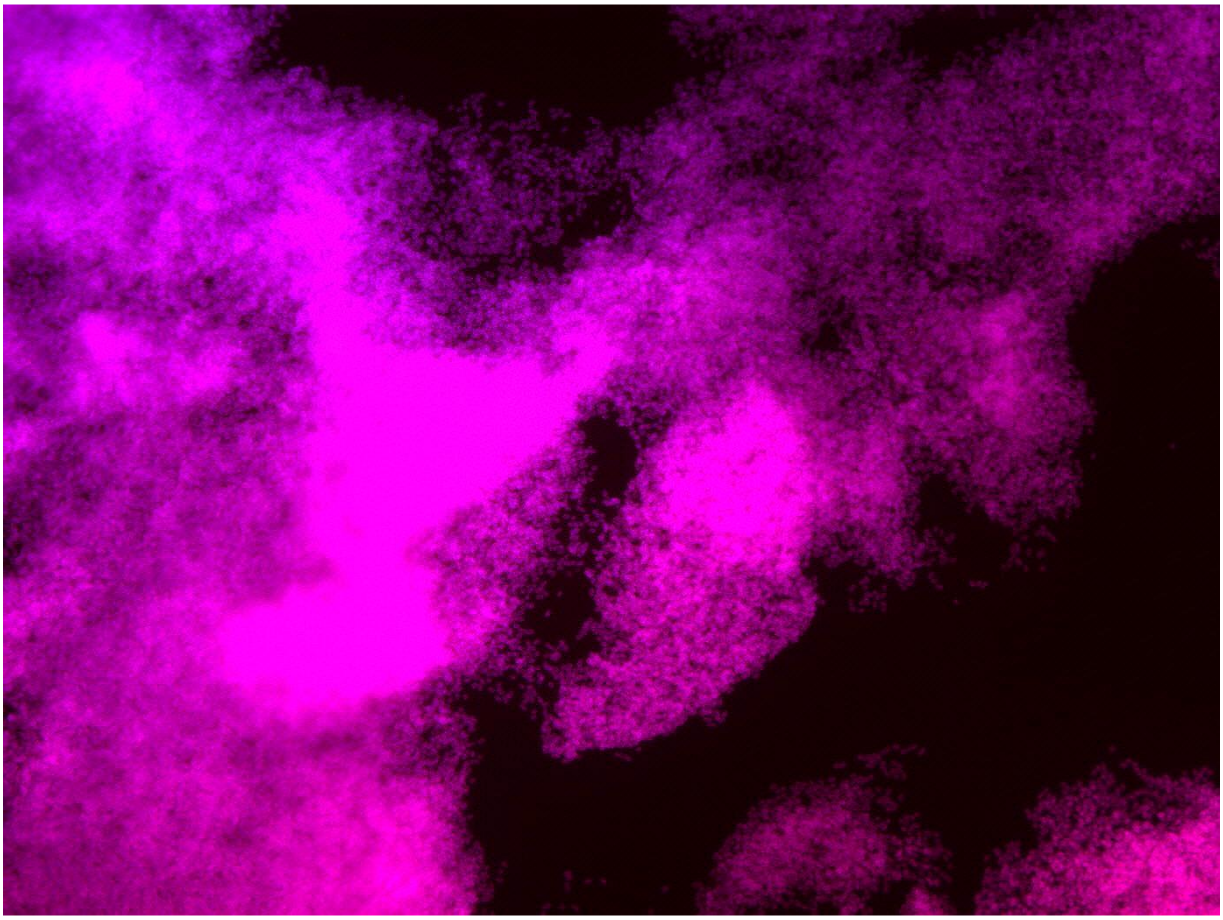

Supplementary Figure 4. Raw FISH image of Figure 2a, day 890, superposition of EUB, PAOmix and GAOmix.

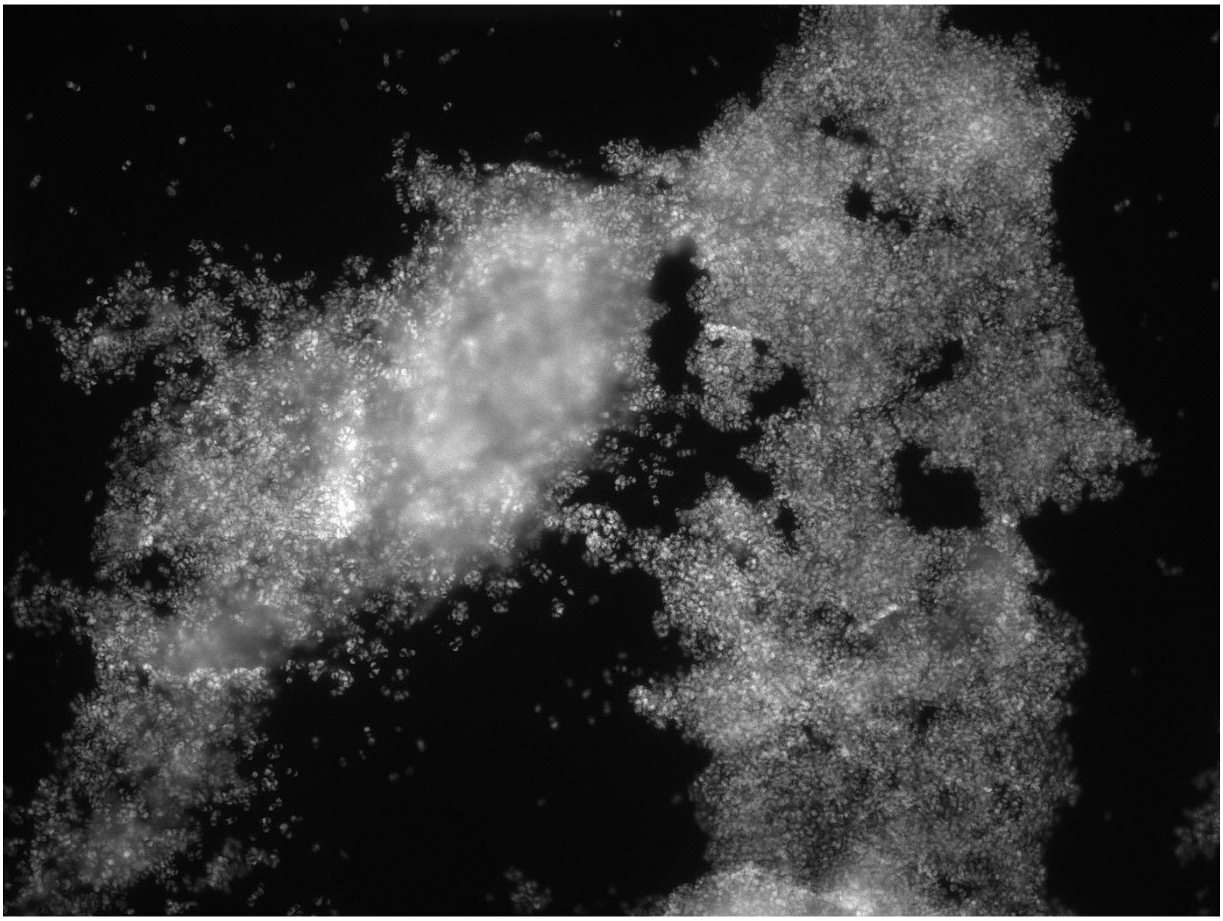

Supplementary Figure 5. Raw FISH image of Figure 2b, day 964, EUB (Cy5).

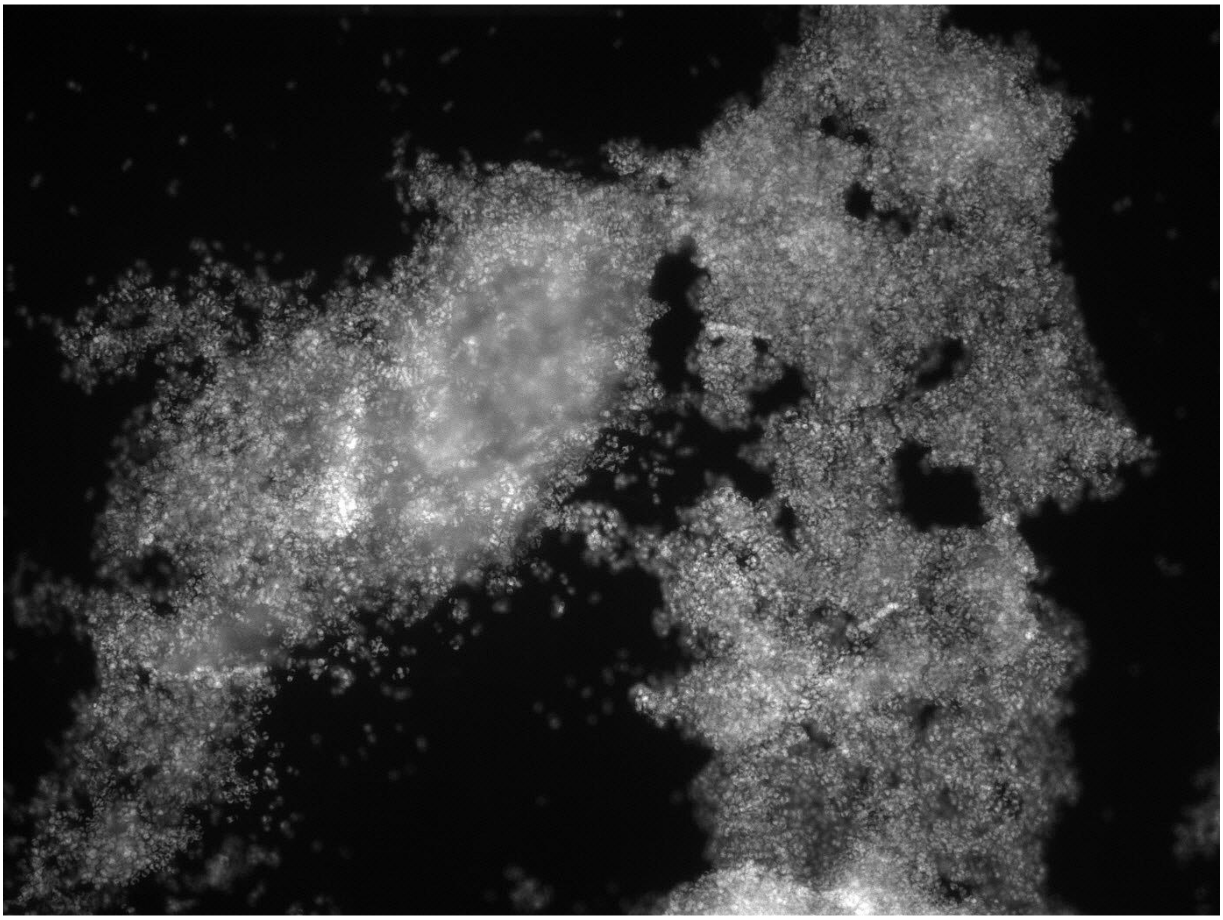

Supplementary Figure 6. Raw FISH image of Figure 2b, day 964, PAOmix (Cy3).

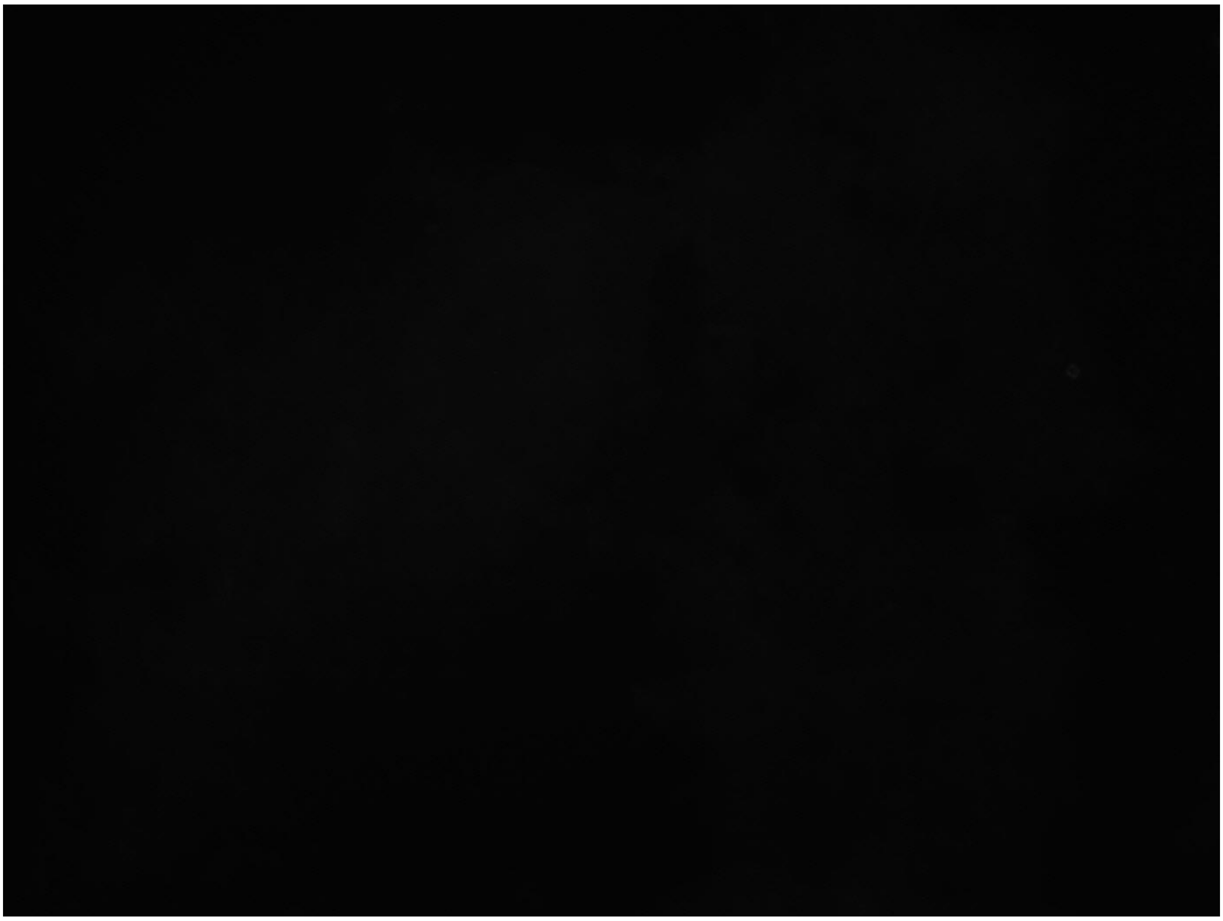

Supplementary Figure 7. Raw FISH image of Figure 2b, day 964, GAOmix (Fluos).

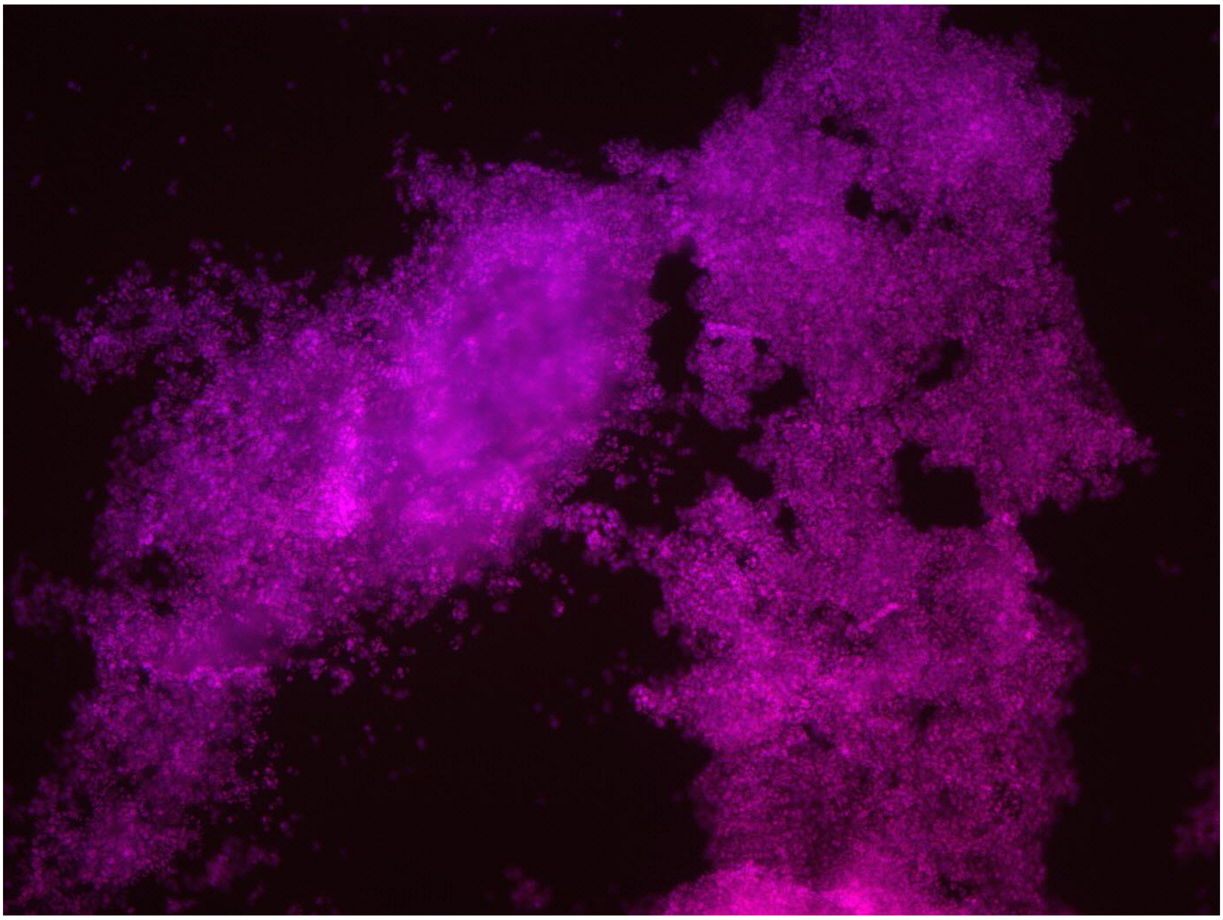

Supplementary Figure 8. Raw FISH image of Figure 2b, day 964, superposition of EUB, PAOmix and GAOmix.

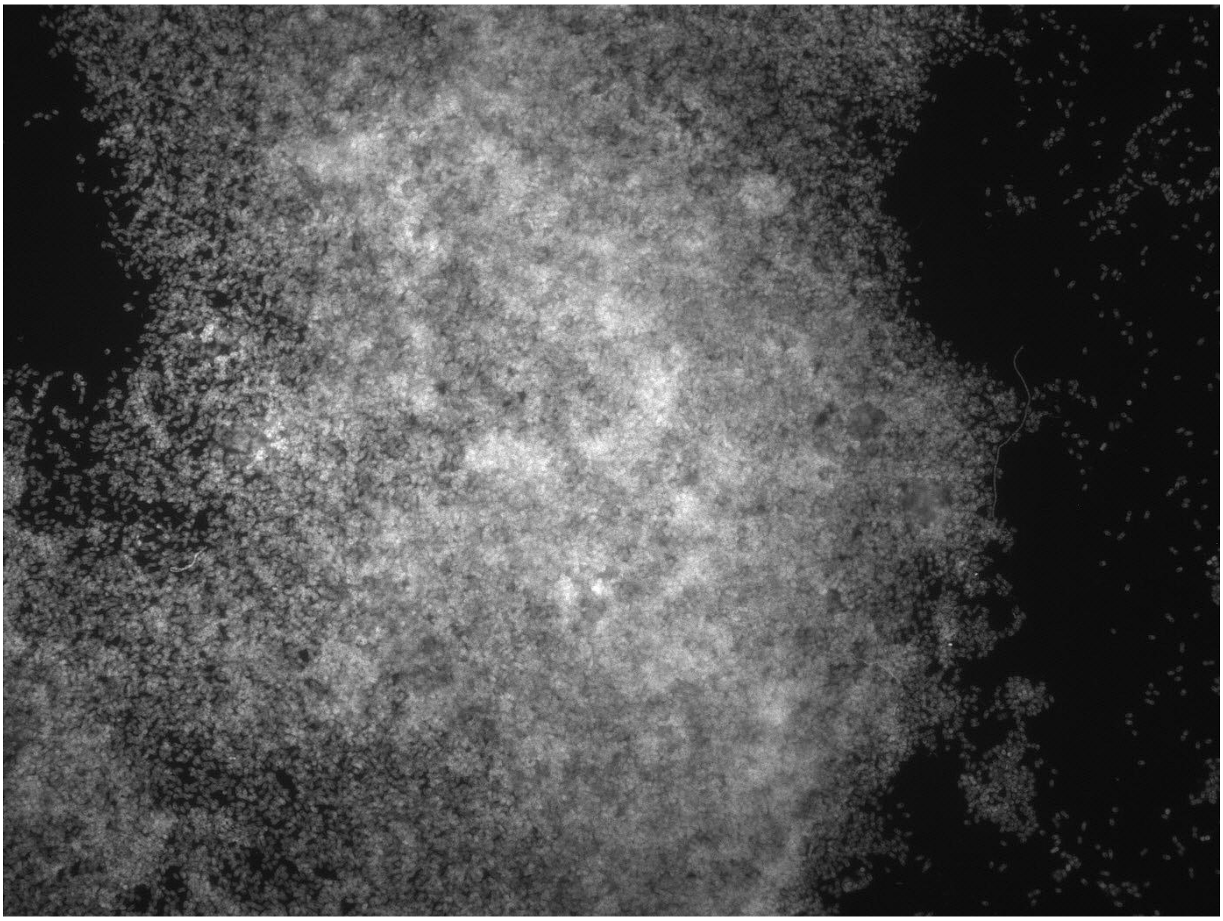

Supplementary Figure 9. Raw FISH image of Figure 2c, day 1034, EUB (Cy5).

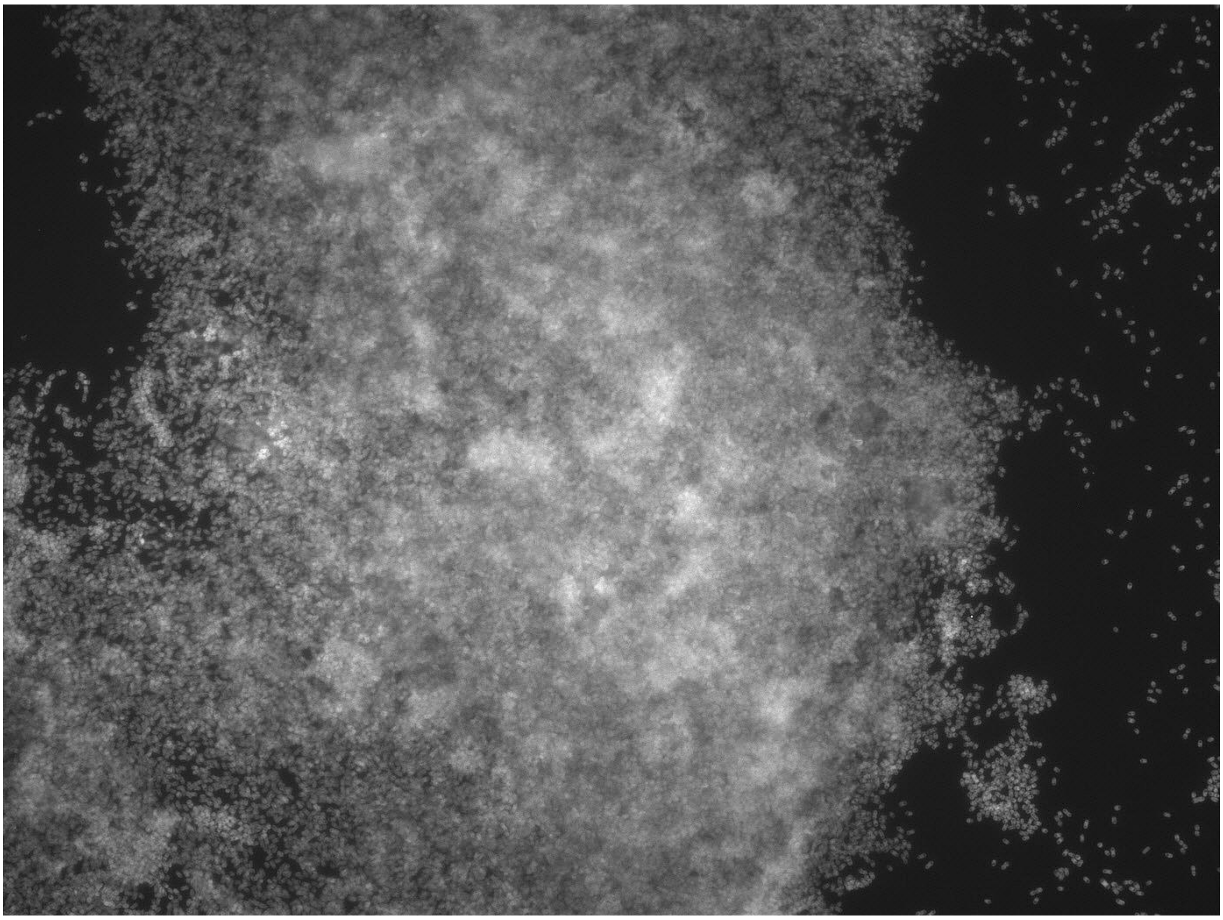

Supplementary Figure 10. Raw FISH image of Figure 2c, day 1034, PAOmix (Cy3).

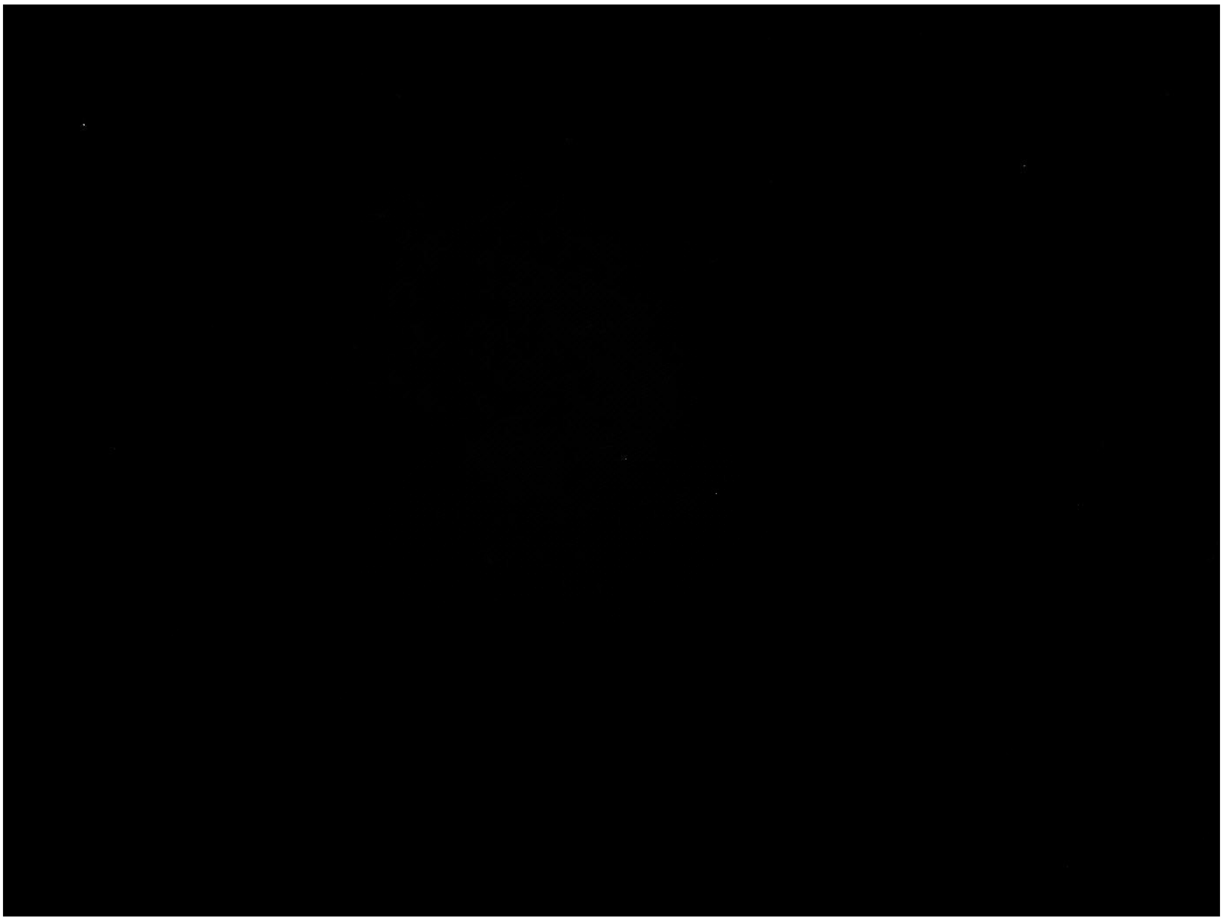

Supplementary Figure 11. Raw FISH image of Figure 2c, day 1034, GAOmix (Fluos).

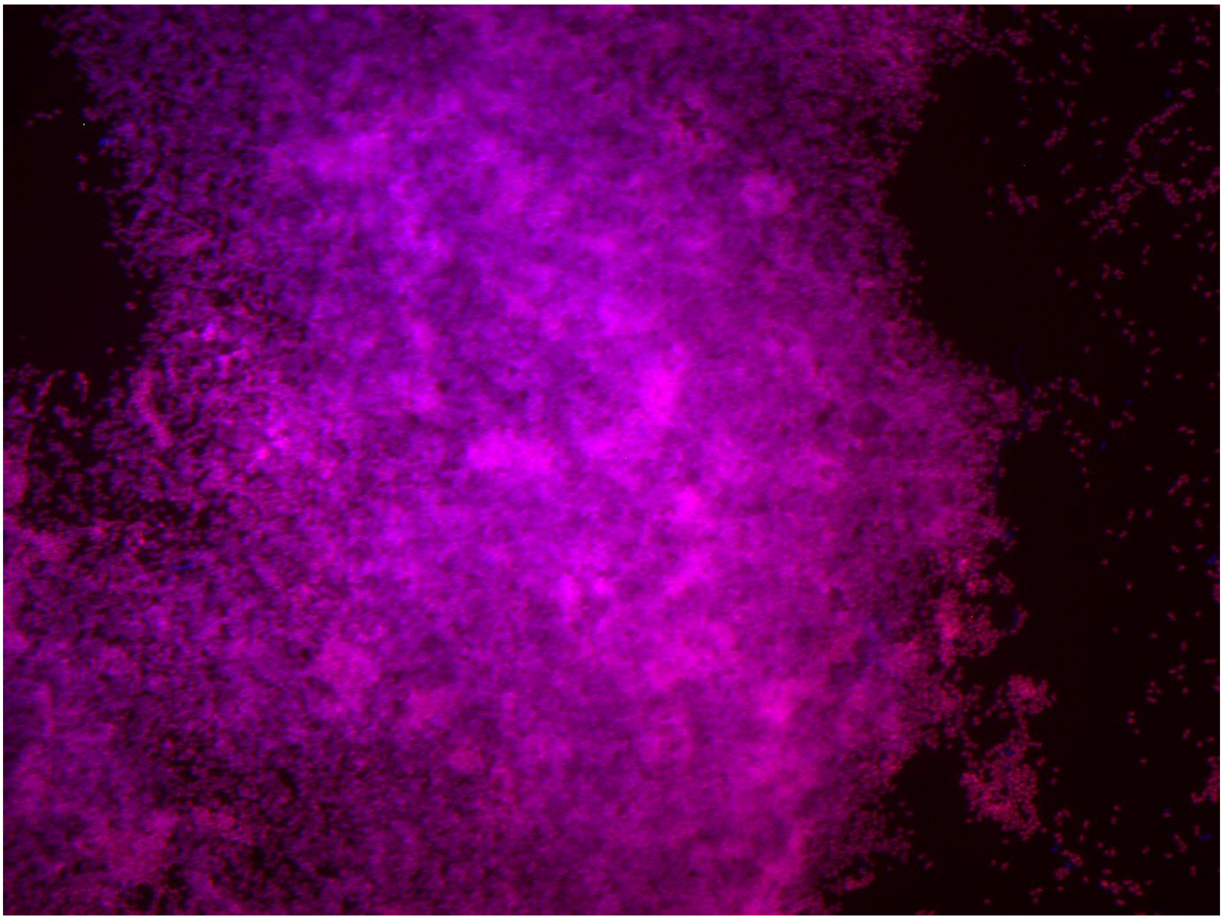

Supplementary Figure 12. Raw FISH image of Figure 2c, day 1034, superposition of EUB, PAOmix and GAOmix.

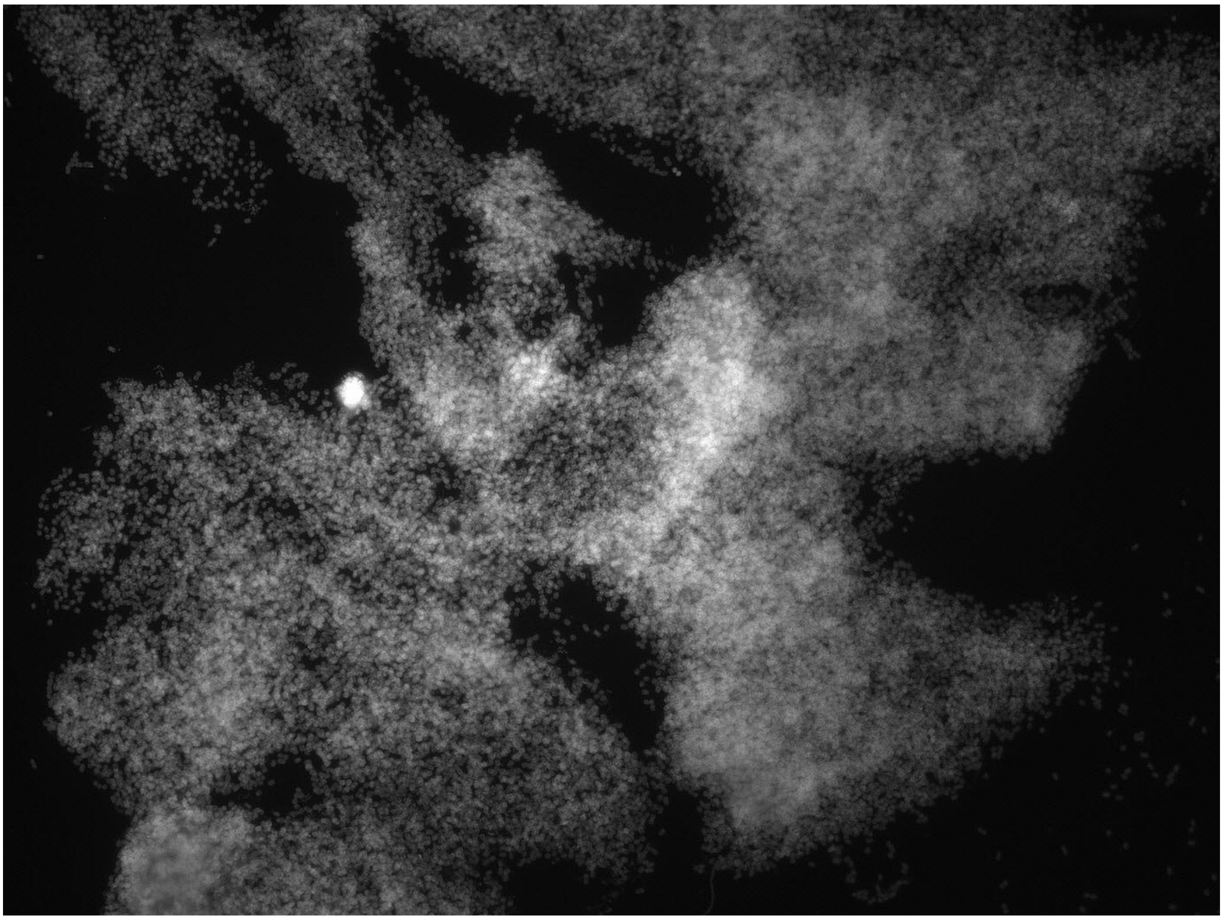

Supplementary Figure 13. Raw FISH image of Figure 2d, day 890, PAOmix (Cy5).

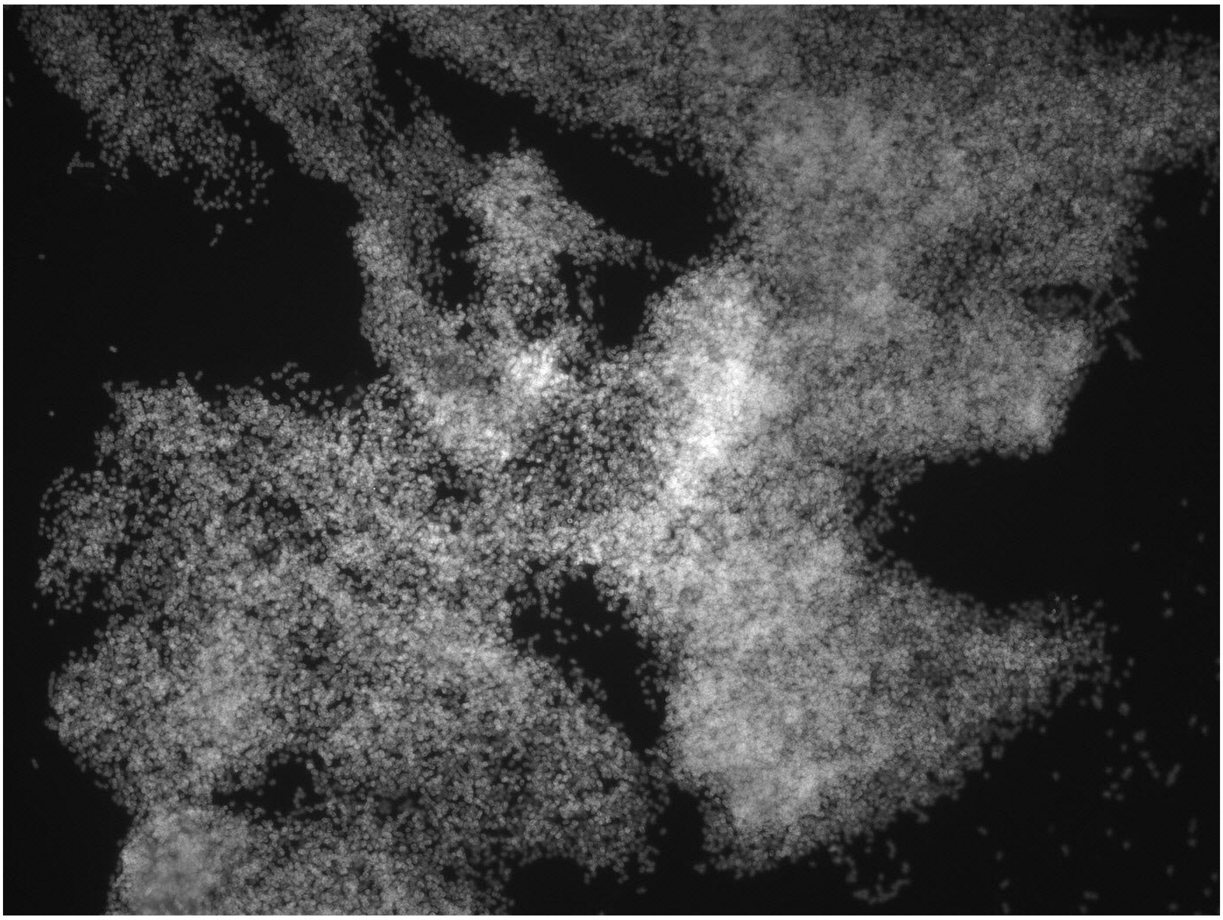

Supplementary Figure 14. Raw FISH image of Figure 2d, day 890, AccII (Cy3).

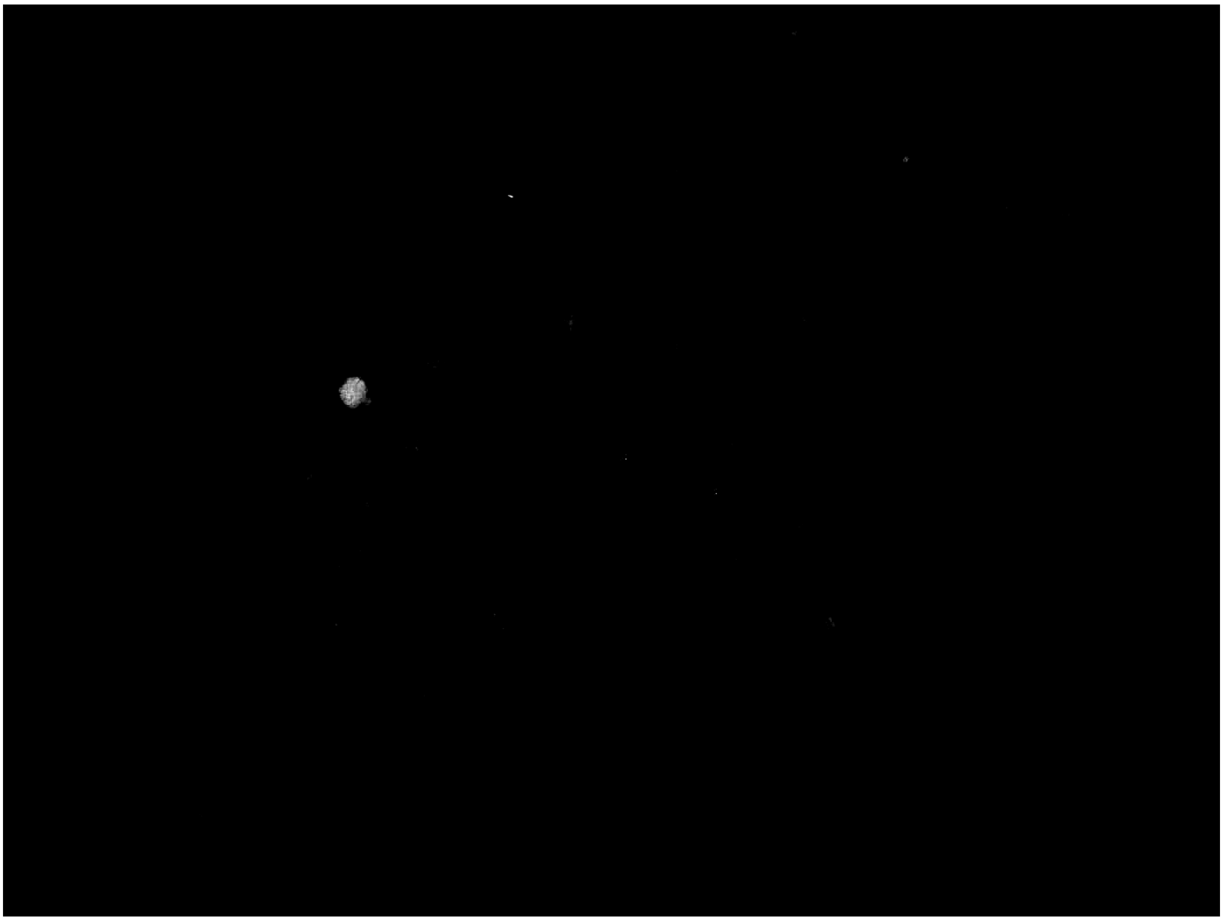

Supplementary Figure 15. Raw FISH image of Figure 2d, day 890, AccI (Fluos).

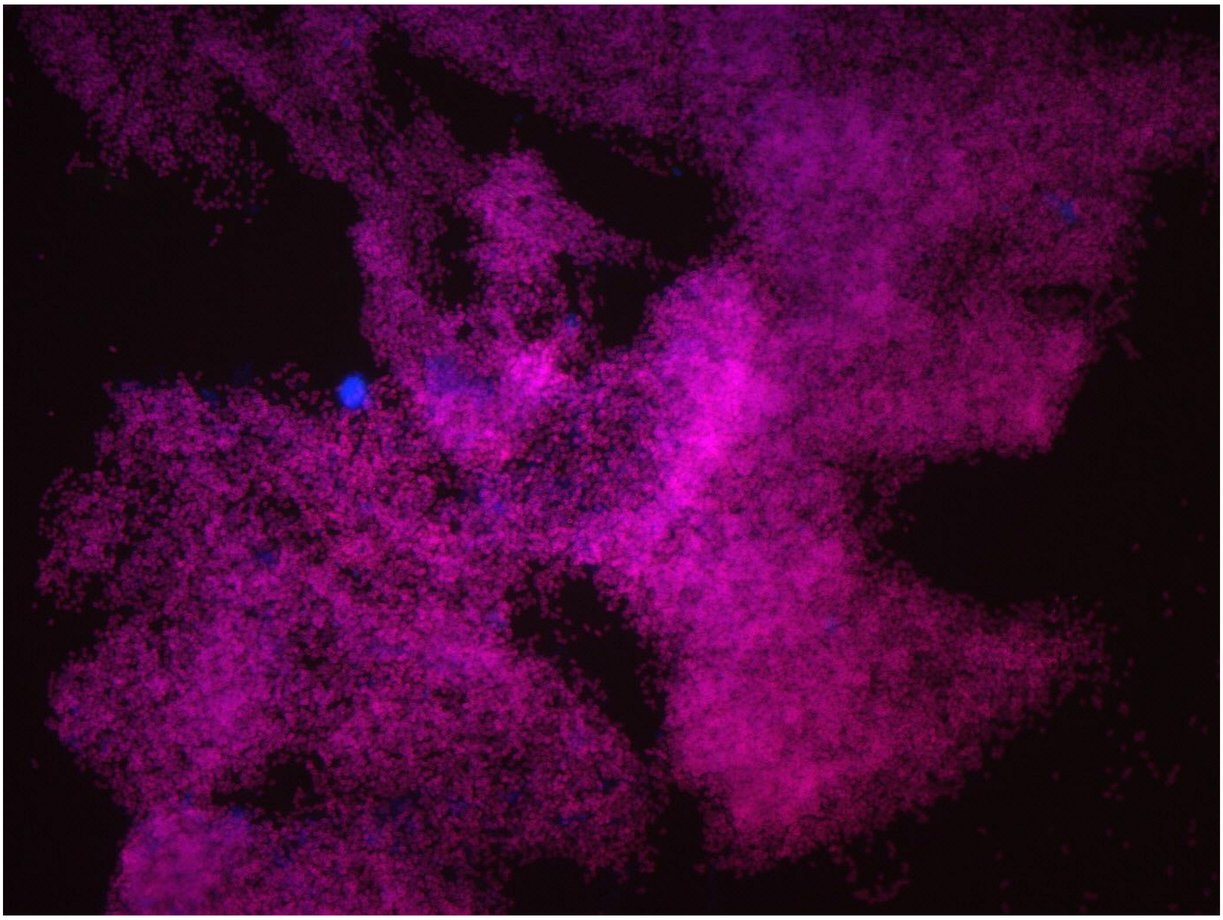

Supplementary Figure 16. Raw FISH image of Figure 2d, day 890, superposition of PAOmix, AccII and AccI.

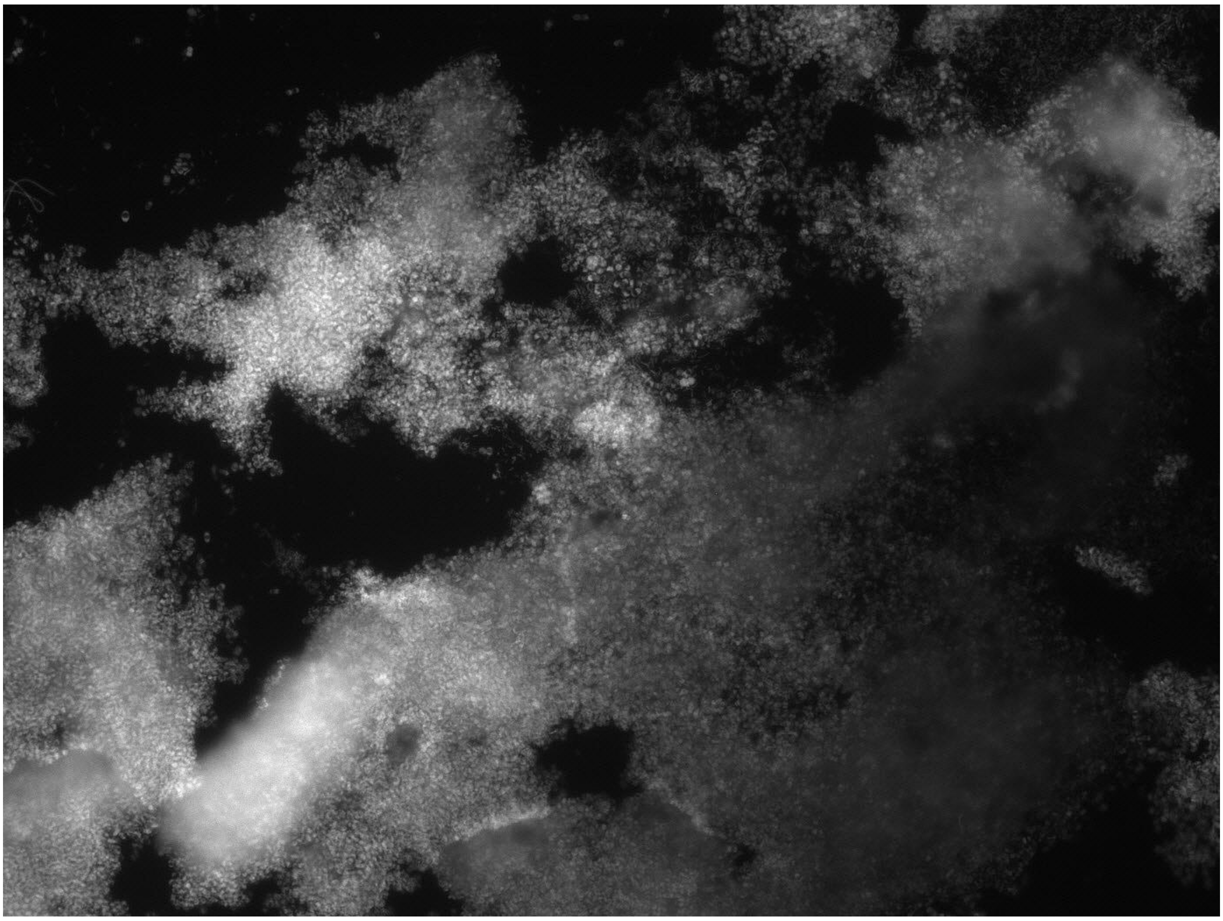

Supplementary Figure 17. Raw FISH image of Figure 2e, day 964, PAOmix (Cy5).

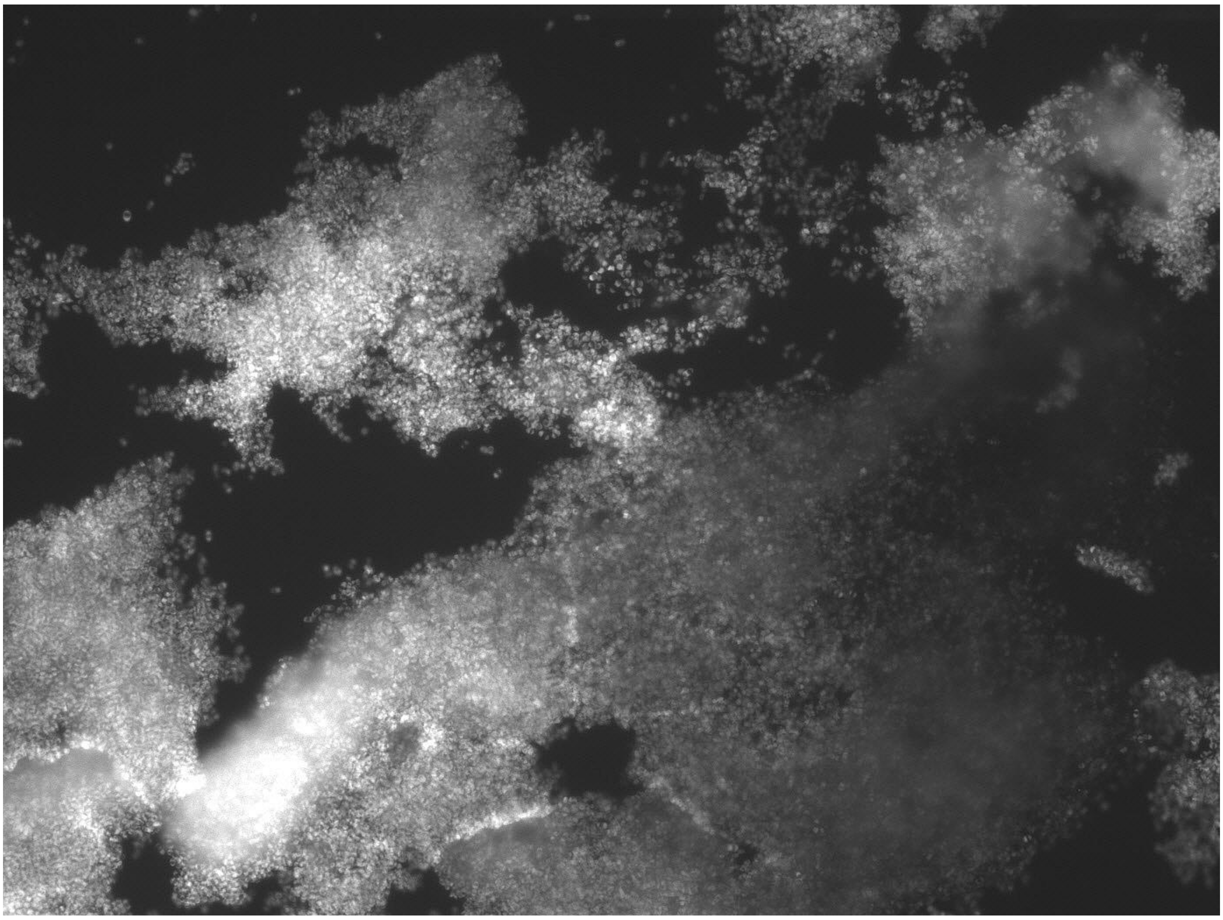

Supplementary Figure 18. Raw FISH image of Figure 2e, day 964, AccII (Cy3).

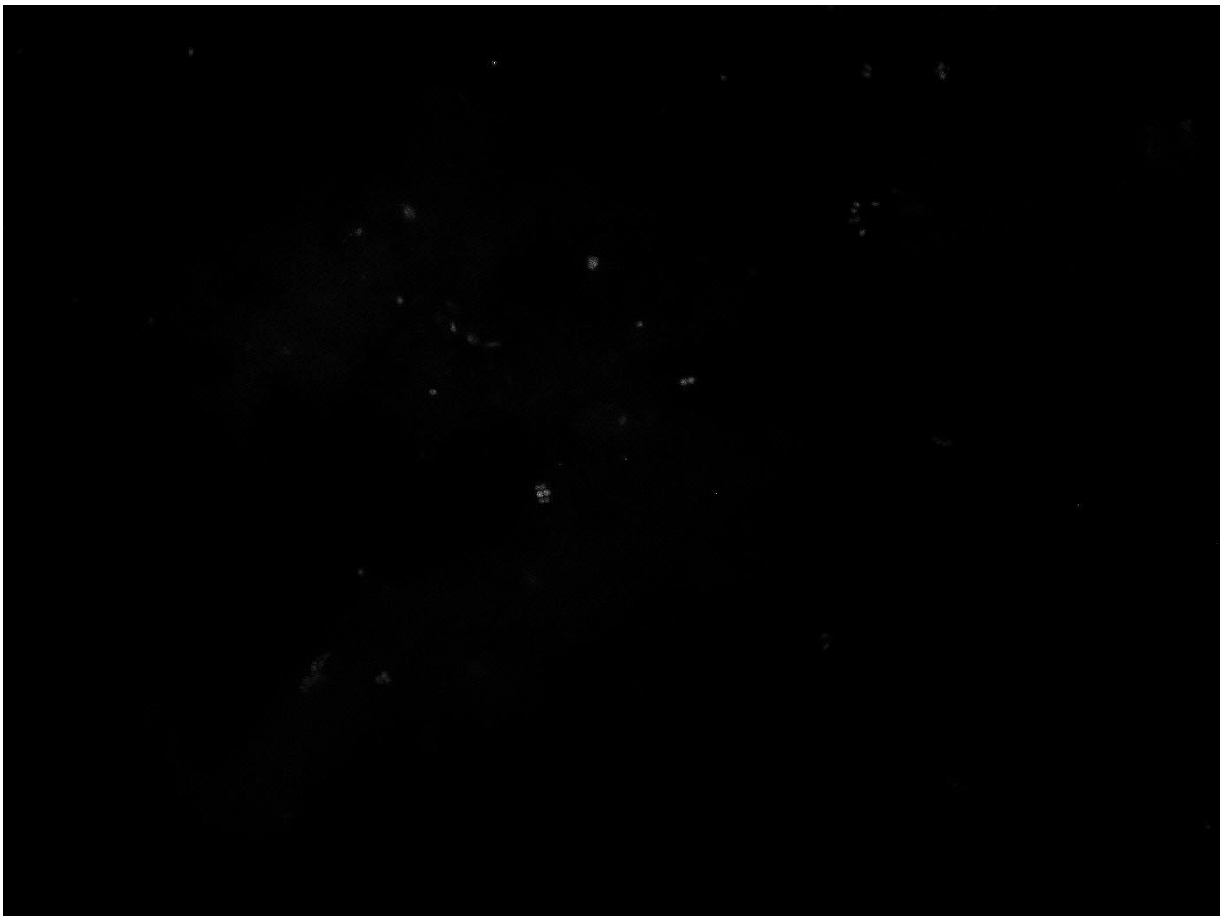

Supplementary Figure 19. Raw FISH image of Figure 2e, day 964, AccI (Fluos)

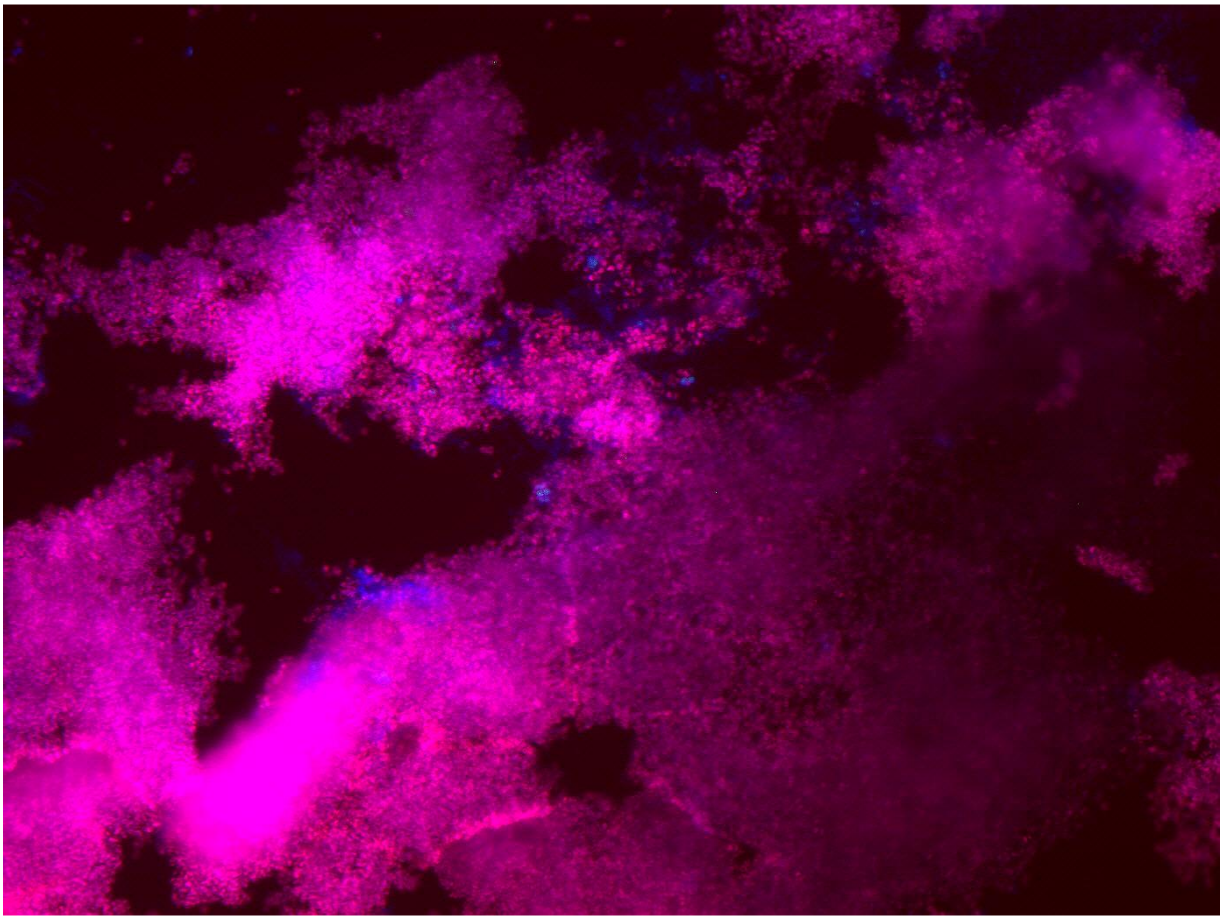

Supplementary Figure 20. Raw FISH image of Figure 2e, day 964, superposition of PAOmix, AccII and AccI.

2f, day 1034

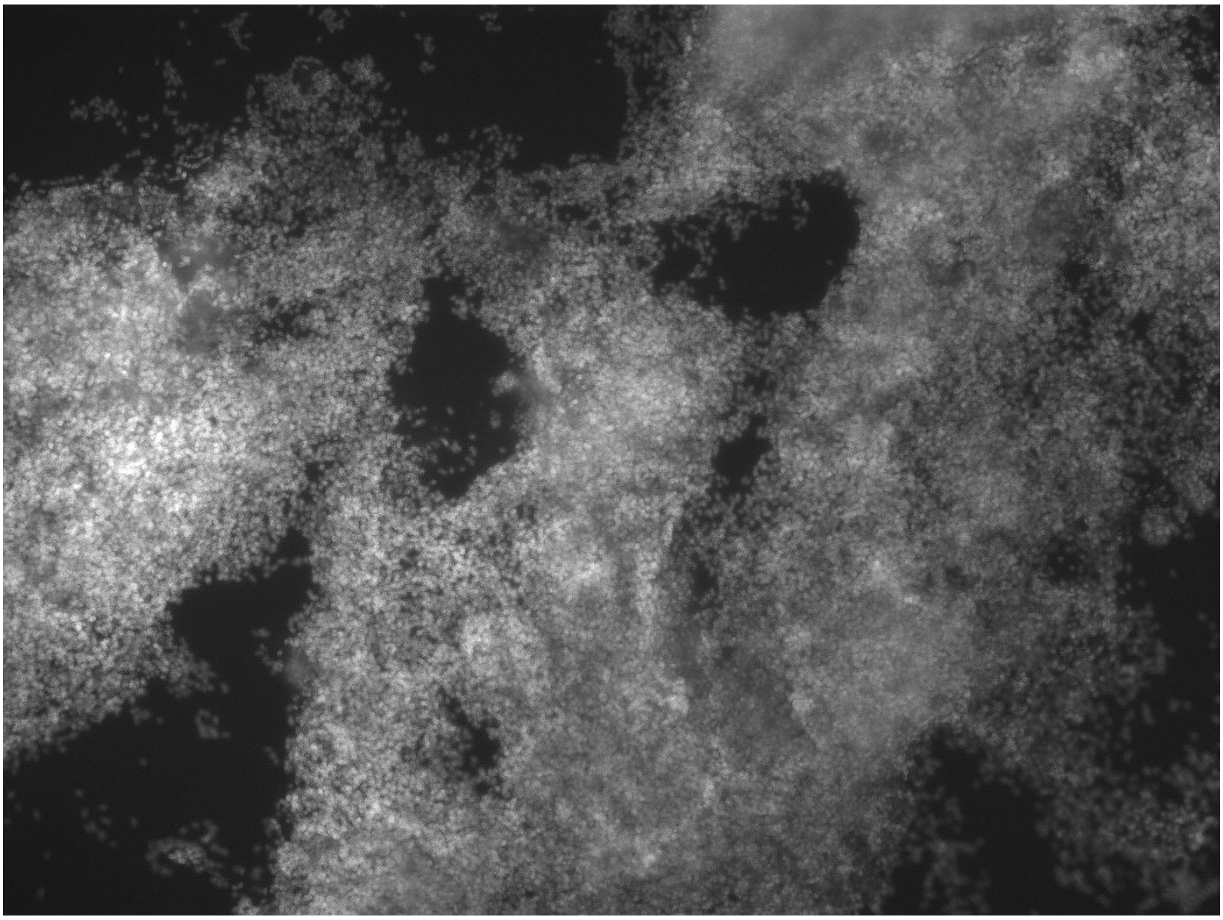

Supplementary Figure 21. Raw FISH image of Figure 2f, day 1034, PAOmix (Cy5).

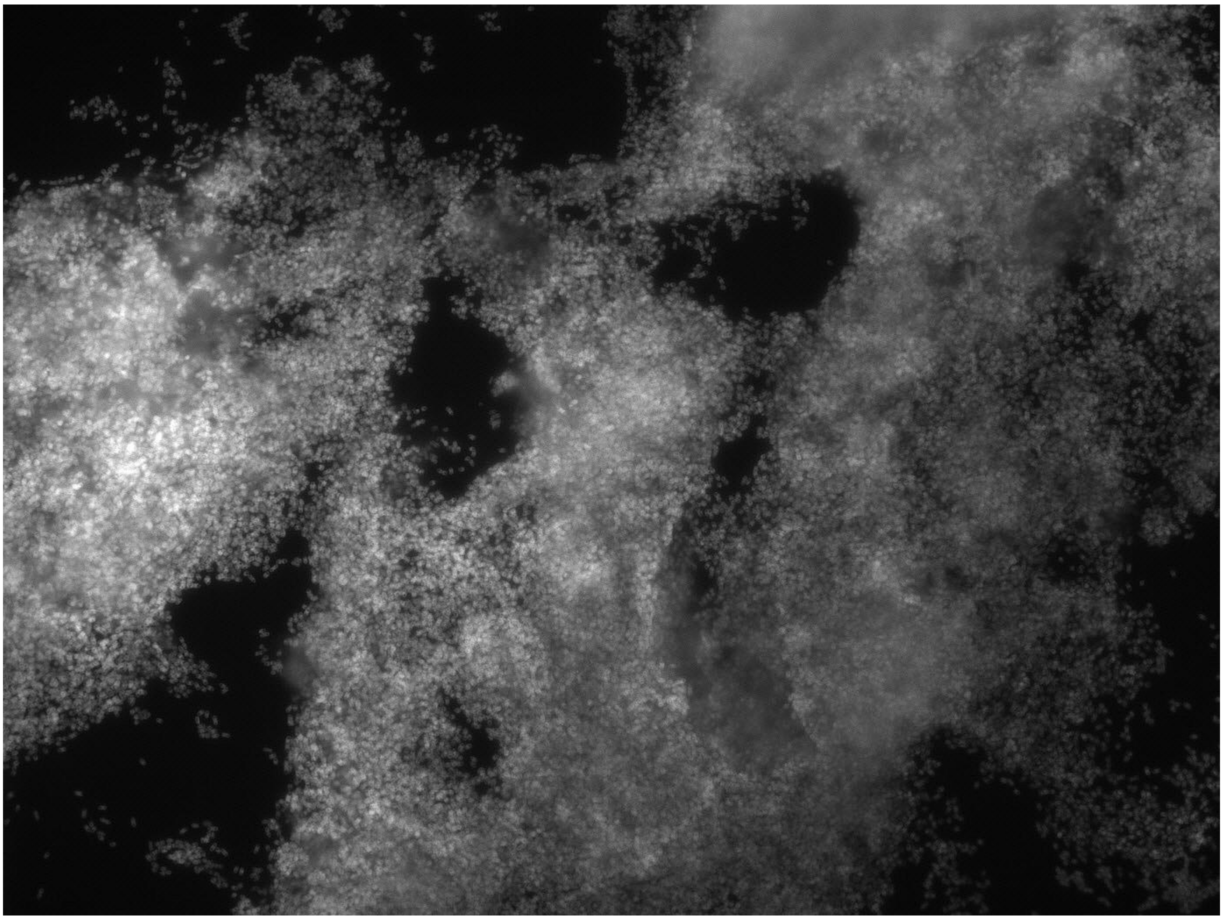

Supplementary Figure 22. Raw FISH image of Figure 2f, day 1034, AccII (Cy3).

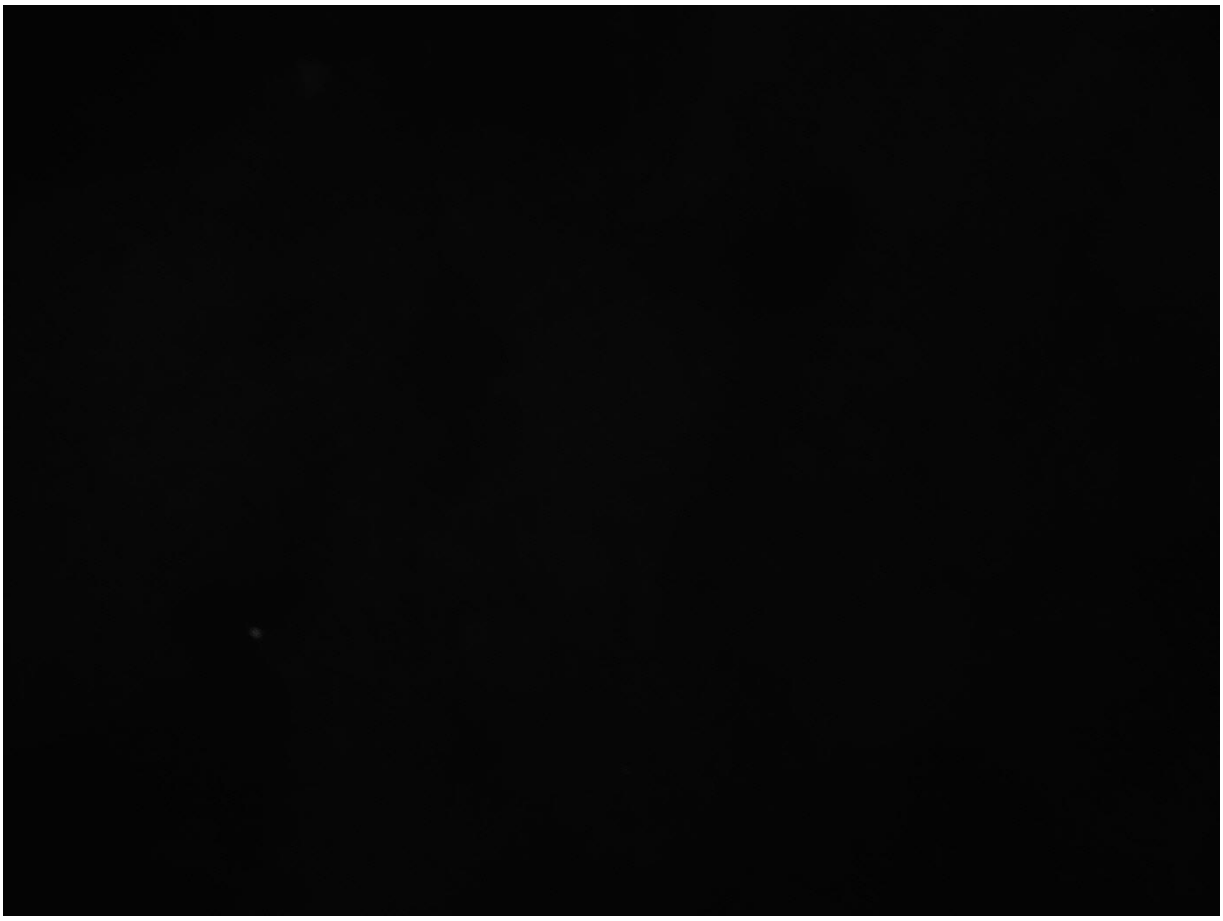

Supplementary Figure 23. Raw FISH image of Figure 2f, day 1034, AccI (Fluos).

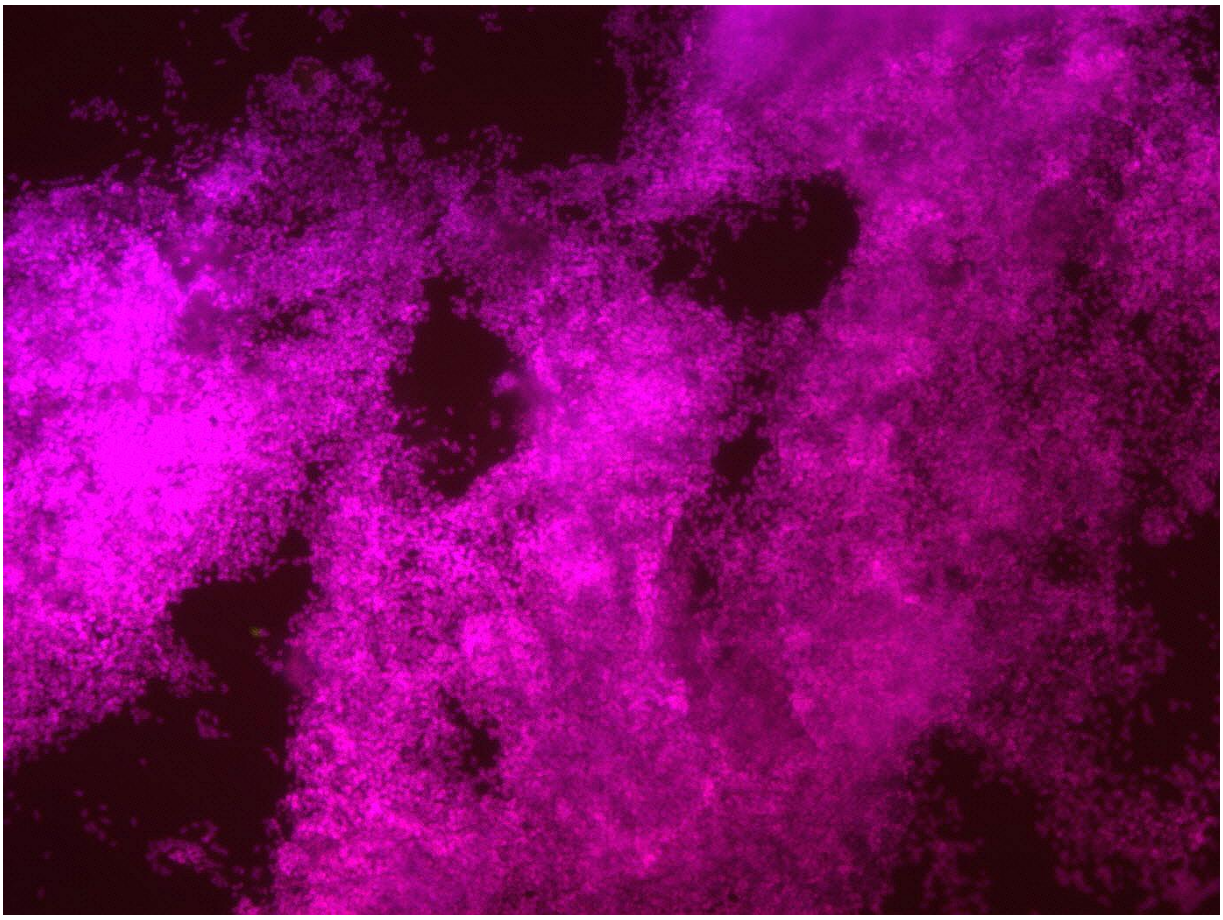

Supplementary Figure 24. Raw FISH image of Figure 2f, day 1034, superposition of PAOmix, AccII and AccI.
